# MnCO_3_-mineralized polydopamine nanoparticles as an activatable theranostic agent for dual-modality imaging-guided photothermal therapy of cancers

**DOI:** 10.7150/thno.77060

**Published:** 2022-09-21

**Authors:** Kyung Kwan Lee, Jae-Hyung Lee, Sang Cheon Lee, Chang-Soo Lee

**Affiliations:** 1 Bionanotechnology Research Center, Korea Research Institute of Bioscience and Biotechnology (KRIBB), Daejeon 34141, Republic of Korea; 2 Department of Life and Nanopharmaceutical Sciences, Graduate School, Kyung Hee University, Seoul 02447, Republic of Korea; 3 Department of Oral Microbiology, School of Dentistry, Kyung Hee University, Seoul 02447, Republic of Korea; 4 Department of Maxillofacial Biomedical Engineering, School of Dentistry, Kyung Hee University, Seoul 02447, Republic of Korea; 5 Department of Biotechnology, University of Science & Technology (UST), Daejeon 34113, Republic of Korea

**Keywords:** cancer, fluorescent polydopamine nanoparticles, mineralization, dual-modality imaging, photothermal therapy, theranostics

## Abstract

**Background:** Single imaging modality is still insufficient to evaluate the biological and anatomical structures of tumors with high accuracy and reliability. Generation of non-specific contrast, leading to a low target-to-background signal ratio, results in low imaging resolution and accuracy. Tumor environment-specific activatable multifunctional contrast agents need to maximize the contrast signals, representing a dual imaging-guided photothermal therapy (PTT) at target tumor sites.

**Methods:** Cellular uptake, cytotoxicity assay, and *in vitro* photothermal conversion efficiency of MnCO_3_-mineralized fluorescent polydopamine nanoparticles (MnCO_3_-FPNPs) were evaluated using 4T1 breast cancer cells. *In vivo* dual-modality imaging was performed using IVIS imaging and a 4.7 T animal MRI systems after injection into 4T1 tumor-bearing nude mice. The effects of photothermal therapeutic through PTT were measured after irradiation with an 808 nm laser (1.5 W/cm^2^) for 10 min, measuring the size of the tumors every 2 days.

**Results:** At physiological pH (7.4), MnCO_3_-FPNP is efficiently quenched. Conversely, at acidic pH (5.4), the strong fluorescence (FL) is recovered due to the dissociation of Mn^2+^ from the FPNPs. At pH 7.4, MnCO_3_-FPNP activity is silenced to enhance water proton relaxation due to unionized MnCO_3_ maintenance; conversely, at acidic pH (5.4), MnCO_3_-FPNPs efficiently release Mn^2+^ ions, thereby resulting in *T*_1_-weighted magnetic resonance (MR) contrast enhancement. MnCO_3_-FPNPs display a promising diagnostic ability for 4T1 breast cancer xenograft models, as well as exhibit a high photothermal conversion efficiency. A successful tumor treatment via their photothermal activity is accomplished within 14 days.

**Conclusions:** Our studies exhibited unique “OFF-ON” activation abilities in FL/MR dual imaging and PTT functions. This approach suggests that the MnCO_3_-FPNPs may serve as a useful platform for various mineralization-based multimodal imaging-guided PTT models for many cancer theranostic applications.

## Introduction

The use of theranostic nanoparticles equipped with the dual functions of diagnosis and therapy has resulted in significant progress in the cancer therapy field 1, 2. They have offered synergistic advantages for cancer treatment in comparison to standard imaging and therapy techniques. Furthermore, theranostic nanoparticles hold great promise in the rapidly growing field of cancer nanomedicine since they can not only be used to detect and monitor cancers at an early stage, but also express on-demand anticancer functions with a high therapeutic efficacy. To date, a number of theranostic agents have been devised through multiple combinations of various imaging modalities and therapeutic tools 3-5. Various imaging techniques, including magnetic resonance (MR), fluorescence (FL) imaging, positron emission tomography (PET), computed tomography (CT), and ultrasound (US) imaging, have been widely adopted for designing theranostic agents 6-9. However, each of the above-mentioned imaging modality possesses its unique strengths and weaknesses and, thus, one single imaging modality is usually not sufficient to evaluate the biological and anatomical structures of tumors with high accuracy and reliability. Therefore, multimodal imaging systems combining two or more complementary imaging modalities have become an attractive strategy for the accurate diagnosis of various tumors.

Of various multimodal imaging modalities, MR-based imaging systems, such as FL/MR, US/MR, CT/MR, and PET/MR, have received intensive interest in the design of promising theranostic nanoparticles, because MR imaging plays a role as a pivotal imaging modality due to its strength including no limitation in tissue-penetrating depth and the acquisition of images with a high spatial resolution 10-14. Among the various MR-based theranostic platforms, the nanoparticles exhibiting FL/MR dual imaging functions are one of the most important research targets due to the complementary synergy of FL and MR imaging. Furthermore, they can display the additional therapeutic function of near-infrared (NIR) light-mediated photothermal activity due to their NIR-absorbing optical property 15, 16. To date, various hybrid contrast agents that can simultaneously improve the contrast enhancement of both FL and MR images have been developed. Most approaches used for the fabrication of these contrast agents are based on superparamagnetic iron oxide nanoparticles (SPIONs)/near-infrared (NIR) dyes, gadolinium (Gd)-integrated nanoparticles/NIR dyes, and manganese-based nanoparticles/NIR dyes 17-19. Although these multimodal contrast agents have shown promising performance by enabling complementary imaging of various tumors, they could not perform as dual activatable probes that can switch the FL/MR dual contrast from an “OFF” state to an “ON” state by sensing specific signals in tumor tissues. Thus, they may generate non-specific contrast, which leads to a low target-to-background signal ratio, resulting in low imaging resolution and accuracy. For this reason, it remains a great challenge to design a tumor environment-specific activatable FL/MR dual contrast agent to minimize the non-specific background signals and, finally, to maximize the contrast signals at target tumor sites.

**Scheme 1:**
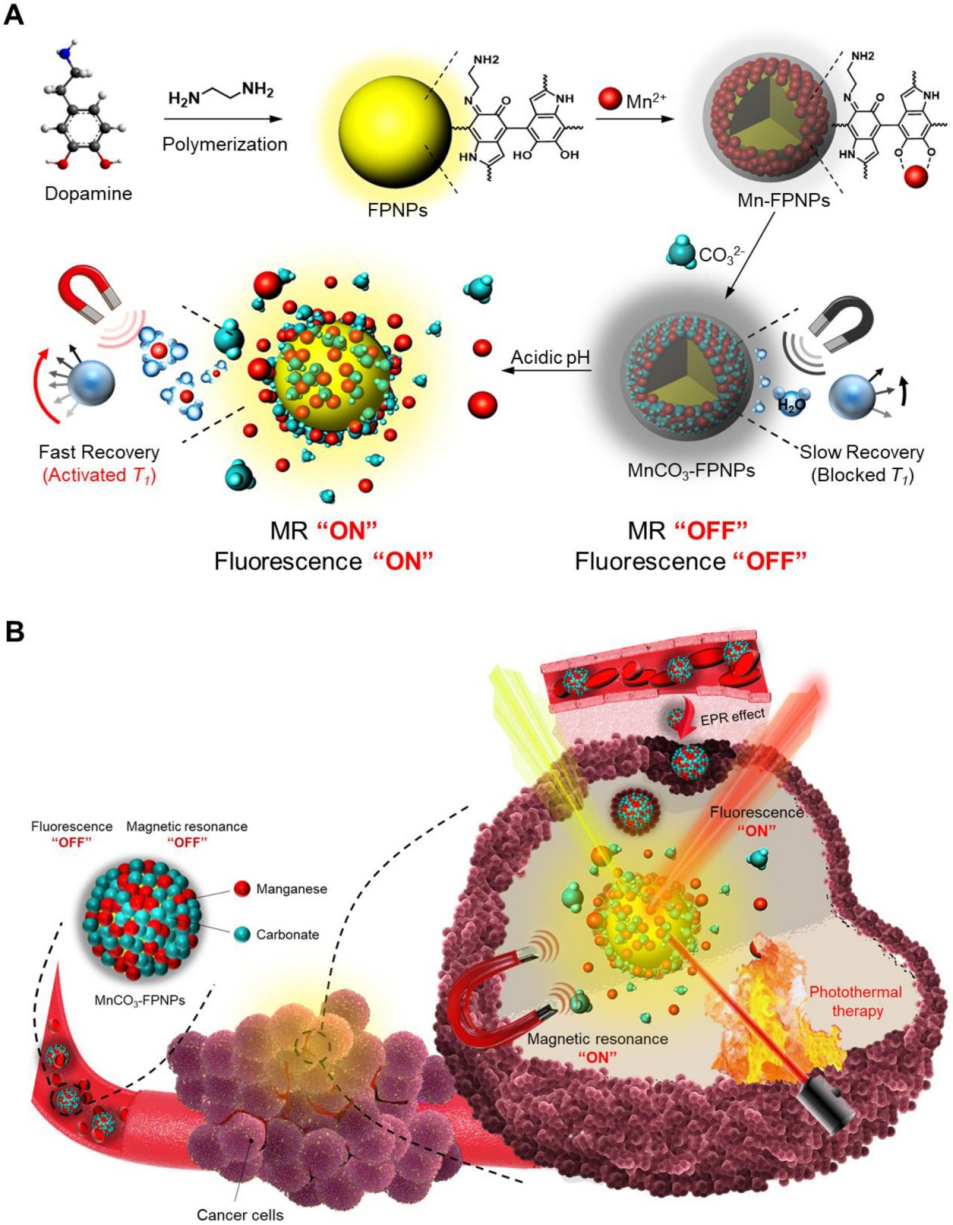
(A) Overall synthetic methods and working principle of MnCO_3_-FPNPs for activatable FL/MR dual-modality imaging. (B) Postulated mechanism of MnCO_3_-FPNPs for FL/MR imaging-guided PTT of tumors after tumoral accumulation by the enhanced permeability and retention (EPR) effect.

Herein, we aim to develop a theranostic MnCO_3_-mineralized polydopamine nanoparticle that has potential for FL/MR dual imaging-guided photothermal therapy (PTT) of cancers. Our key strategy is to develop MnCO_3_-mineralized spherical fluorescent polydopamine nanoparticles (MnCO_3_-FPNPs) as activatable theranostic agents, which not only allow a dual FL/MR “OFF to ON” signal in response to the intracellular acidic pH of tumors, but also exhibits dual imaging-guided photothermal therapeutic functions. **Scheme 1** shows the overall process of fabrication of the MnCO_3_-FPNPs and how they are activated for dual FL/MR imaging in tumor cells and further display photothermal activities for tumor treatment. In brief, for the preparation of the MnCO_3_-FPNPs, Mn^2+^ ions were first bound to the surface of the FPNPs via metal ion-catechol chelation affinity (**Scheme 1A**).This process leads to quenching of the FPNP fluorescence. The further addition of CO_3_^2-^ ions forms electric double layers, and finally induces ionic supersaturation on the FPNPs, leading to the deposition of crystalline MnCO_3_ minerals on the FPNPs. The resulting MnCO_3_-FPNPs are in an inactivated state (“OFF” state), being ready for FL/MR dual imaging and to have a photothermal function. On the other hand, under an endosomal acidic pH range, the MnCO_3_-FPNPs are activated to simultaneously enhance the dual FL/MR contrast (“ON” state) due to MnCO_3_ dissolution. Moreover, under dual FL/MR imaging acquisition, the simultaneous near-infrared (NIR) irradiation enables the MnCO_3_-FPNPs to exhibit photothermal functions by absorbing NIR light and converting it into heat for tumor ablation (**Scheme 1B**).

We place a special emphasis on the role of MnCO_3_ mineralization on the surface of FPNPs on these unique “OFF-ON” activation mechanisms of dual imaging and the PTT functions of the MnCO_3_-FPNPs, as follows. First, the polydopamine (PDA)-based nanoparticles have played significant roles in cancer therapy/imaging due to their unique characteristics, such as their universal metal-binding affinity and fluorescent functions 20-27. However, the existing PDA-based nanoparticles lack the activatable switch of the fluorescence from an “OFF” to “ON” state. In our work, we aimed to develop FPNPs with the pH-activatable “OFF-ON” FL imaging function through Mn^2+^ chelation and MnCO_3_ mineralization on the FPNPs. In detail, at physiological pH, the quenched state of the FPNP fluorescence is maintained, whereas at acidic pH in tumor cells, the dissociation of MnCO_3_ minerals from the FPNPs results in the recovery of the fluorescence of the FPNPs. Second, the MnCO_3_ mineralized on the FPNPs has a stable nonionic crystalline structure at neutral pH and, thus, its MR activity is dormant at physiological pH because MnCO_3_ does not generate MR-active Mn^2+^ ions due to the extremely low aqueous solubility of MnCO_3_ at neutral pH. It is noteworthy that when the pH decreases, the aqueous solubility of MnCO_3_ increases 28. Hence, at tumoral acidic pH, the MnCO_3_ on the FPNPs is ionized to produce a number of Mn^2+^ ions, which enhance water proton relaxation to generate a strong *T*_1_-weighted MR contrast enhancement. Third, the deposition of MnCO_3_ on the surface of the FPNPs enables the MnCO_3_-FPNPs to absorb NIR light because the MnCO_3_ can change the energy band gap of the FPNPs. Consequently, this NIR-absorbing property of the MnCO_3_-FPNPs can exhibit PTT functions. In this work, we demonstrate that the rationally designed MnCO_3_-FPNPs can display cancer-specific dual imaging-guided therapeutic effects through *in vitro* and *in vivo* proof-of-concept studies.

## Results and Discussion

### Synthesis and characterization of MnCO_3_-FPNPs

**Figure 1:**
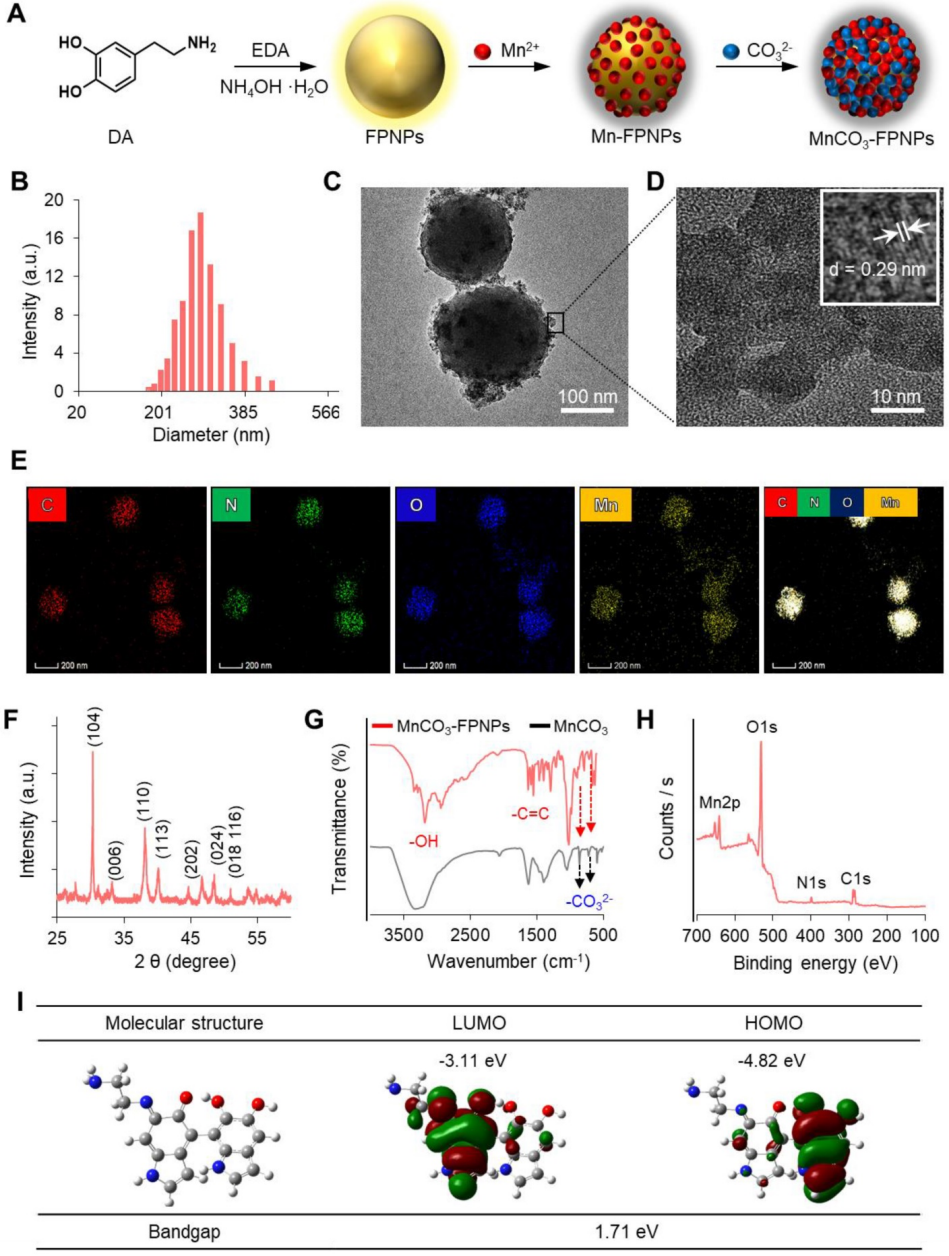
(A) The synthetic method used for the MnCO_3_-FPNPs. Structure characterization of the MnCO_3_-FPNPs using (B) Dynamic light scattering (DLS) and (C), (D) TEM. (E) Energy-dispersive X-ray spectroscopy (EDS) mapping images of the MnCO_3_-FPNPs, including carbon (C), nitrogen (N), oxygen (O), and manganese (Mn) (HAADF: High-angle annular dark-field TEM image). (F) X-ray diffraction (XRD) spectrum of the MnCO_3_-FPNPs. (G) FT-IR spectra of the MnCO_3_-FPNPs and MnCO_3_. (H) X-ray photoelectron spectroscopy (XPS) survey scan spectrum of the MnCO_3_-FPNPs. (I) Theoretical calculated molecular orbitals of FPNPs using DFT calculations at the B3LYP/3-31G (d,p) level.

As the prototype of the MnCO_3_-FPNPs, we previously synthesized fluorescent PDA nanoparticles (FPNPs) using DA and ethylenediamine (EDA) in a Tris-HCl buffer solution for bioimaging 29. The prototype of FPNPs showed green fluorescence emission, biocompatibility, a worm-like shape with a diameter of several tens of nanometers, and an irregular structure. However, for tumor accumulation via the enhanced permeability and retention effect, the nanoparticles should have a uniform shape and a suitable size of approximately 20-300 nm 30-32. In this study, by using an aqueous solution of ammonia (NH_4_OH) as a reaction medium, we have successfully prepared spherical fluorescent PDA nanoparticles (FPNPs) with a highly uniform shape and yellow fluorescence emission that can be used as cancer theranostic agents. The synthetic procedure used for preparing the MnCO_3_-FPNPs is illustrated in **Figure 1A**. In brief, DA HCl and EDA were simultaneously added to an aqueous NH_4_OH solution under vigorous stirring for 12 h. After polymerization, the resulting light-brown solution was treated with an aqueous MnCl_2_ solution. After the binding of the Mn^2+^ ions to the surface of the FPNPs through catechol-metal coordination, the CO_3_^2-^ ions were added to the Mn-FPNP solution to finally produce the MnCO_3_-mineralized FPNPs. The zeta potential of each synthetic step was 2.85, -2.01, and 0.17 mV for the FPNPs, Mn-FPNPs, and MnCO_3_-FPNPs, respectively (**Figure S1**). Morphologically, the control PDA nanoparticles, the FPNPs, and the MnCO_3_-FPNPs have a spherical shape with a narrow size distribution, and could be synthesized more uniformly than the previously reported prototype of FPNPs (**Figure S2**). The MnCO_3_-FPNPs show a size distribution in the 190 to 370 nm range (**Figure 1B**). As revealed by transmission electron microscopy (TEM) imaging, the morphology of the MnCO_3_-FPNPs is spherical with an average diameter of 230 nm. Moreover, in contrast with the FPNPs, the MnCO_3_-FPNPs have a virus-like shape due to the formation of MnCO_3_, which is exposed on their surface (**Figure 1C**). The lattice fringe spacing found on the surface of the MnCO_3_-FPNPs was 0.29 nm, belonging to the crystal planes of the MnCO_3_ in the vertical direction (**Figure 1D**) 33. This finding indicates the successful mineralization of crystalline MnCO_3_ on the surface of the FPNPs. TEM-associated energy-dispersive X-ray spectroscopy (EDS) mapping images were captured to assess the atomic compositions of the MnCO_3_-FPNPs (**Figure 1E**). Powder X-ray diffraction (XRD) was carried out to reveal the crystallographic phase structure of the MnCO_3_-FPNPs (**Figure 1F**). As a result, the pattern associated with MnCO_3_ (JCPDS No. 44-1472) was matched only to that of MnCO_3_-FPNPs, but not to that of PDA, FPNPs, or Mn-FPNPs (**Figure S3**). FT-IR spectroscopic analyses showed C=C and O-H stretching vibration bands at 1,628 cm^-1^ and 3,380 cm^-1^, respectively, which are ascribed to the PDA structures (**Figure 1G**). In addition, the C-O vibration peaks of CO_3_^2-^, which are originated from MnCO_3_, are not found in the PDA, FPNPs, or Mn-FPNPs, whereas the MnCO_3_-FPNPs showed those peaks at 884 cm^-1^ and 717 cm^-1^ (**Figure S4**) 34.

X-ray photoelectron spectroscopy (XPS) analyses were performed to identify the elemental compositions and chemical bonds of the PDA, FPNPs, Mn-FPNPs, and MnCO_3_-FPNPs. The survey scan spectrum of the MnCO_3_-FPNPs demonstrates that the MnCO_3_-FPNPs are composed of carbon, nitrogen, oxygen, and manganese elements (**Figure 1H**). **Figure S5** also shows the MnCO_3_-FPNPs elemental composition changes at each synthetic step. For a more accurate investigation of the MnCO_3_ mineralization on the Mn-FPNPs, we found that the peak assigned to Mn^2+^ was observed in both the Mn-FPNPs and the MnCO_3_-FPNPs at 642 eV, whereas the peaks assigned to CO_3_^2-^, which are associated with MnCO_3_, were found only in the MnCO_3_-FPNPs, at 289 eV (**Figure S6**) 35, 36. These results indicate that the mineralization of MnCO_3_ successfully occurred on the surface of the FPNPs. We also performed density functional theory (DFT) calculation to determine the energy of the FPNPs by EDA doping (**Figure 1I**). The highest occupied molecular orbital (HOMO) and the lowest unoccupied orbital (LUMO) of FPNPs were calculated to be - 4.82 eV and -3.11 eV, respectively, and the bandgap of FPNPs was 1.71 eV. These results indicate the structural change of PDA due to the addition of EDA. The HOMO-LUMO energy level of the FPNP was clearly distinct from that of the conventional PDA (**Figure S7**) 37.

### pH-dependent fluorescence quenching and recovery

The FPNPs, which are synthesized by adding EDA during PDA polymerization under a weak alkaline solution, were found to exhibit fluorescence properties. As discussed in our previous report for the fluorescent FPNPs 29, the reaction between the EDA and PDA particles seems to be a key factor for such luminescent features of the FPNPs because the PDA particles prepared without EDA are non-emissive in the UV/vis wavelength range. The most noticeable feature is that the photophysical property of the FPNPs is dependent on the time of addition of EDA (a nucleophilic monomer) during the PDA polymerization reaction. When EDA and DA were simultaneously added, the resulting fluorescent particles exhibited yellow fluorescence with a *λ*_max_ of 560 nm. In contrast, the fluorescence tended to undergo a blue shift as the addition of EDA was delayed (**Figure S8**). It is well known that the lone pair electrons of the amine groups could be donated to the conjugated structure of the fluorescent nanoparticles, such as in the case of amine-functionalized graphene quantum dots. The resulting increased electron density lowers the band gap of the fluorescent nanoparticles, thereby resulting in a red shift 38. When EDA and DA are simultaneously added at the initial reaction state, the EDA can participate in the diverse reaction steps from the molecular to the nanoparticle state. The numerous amine groups from EDA may exist from the core to the surface areas of the resulting FPNPs. As expected, the delayed addition of EDA probably results in FPNPs with the majority of the EDA-originated amine groups on their surfaces. Thus, it is likely that the FPNPs with a larger amount of the amine groups originated from EDA more strongly alter their electronic energy levels and consequently cause a more pronounced red-shift of their fluorescence spectra. We chose the FPNPs prepared via simultaneous addition of EDA and DA for estimation of the optical property and for the *in vivo* imaging studies.

**Figure 2:**
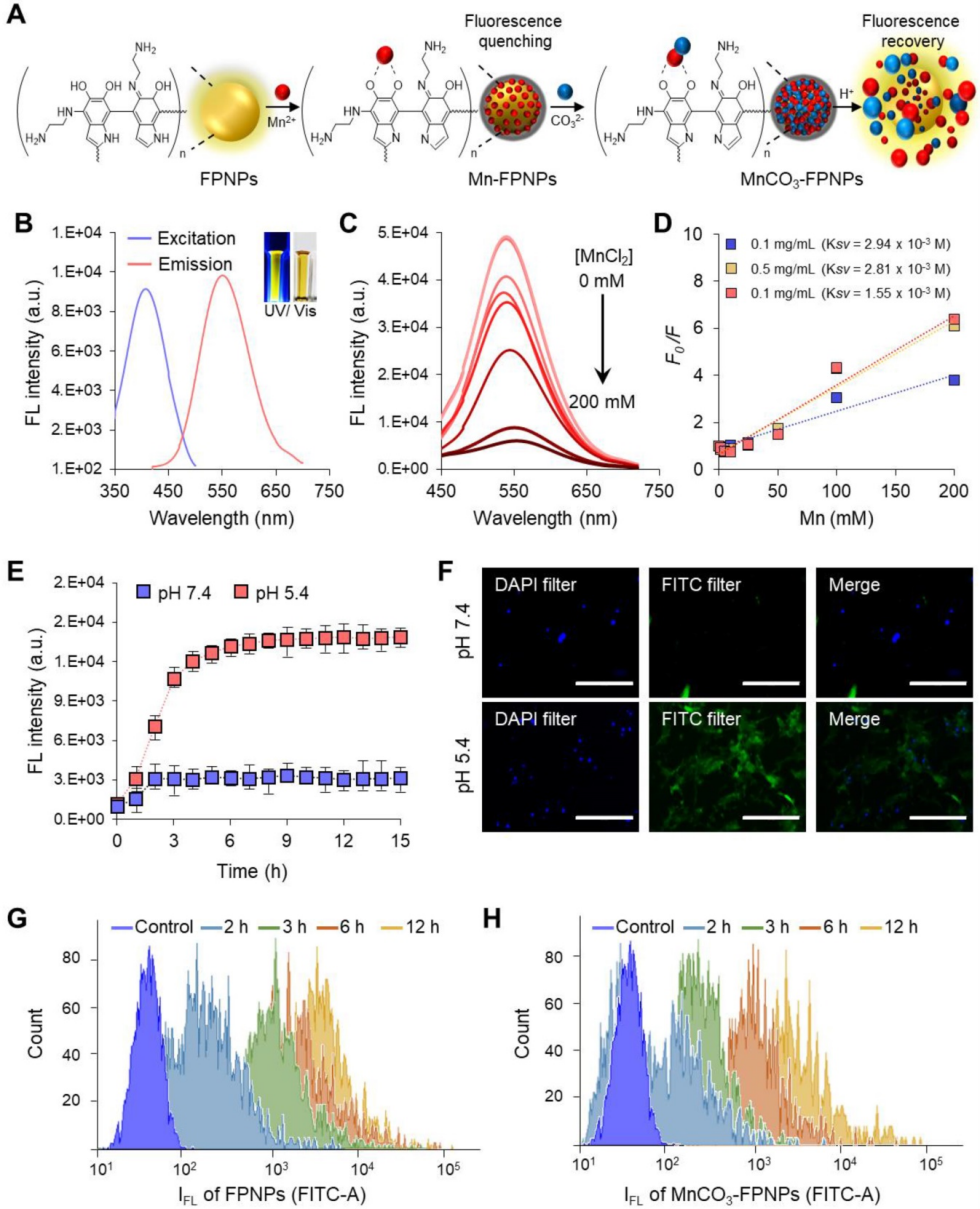
(A) Schematic illustration of the FL “OFF to ON” working process of MnCO_3_-FPNPs. (B) Excitation and emission spectra of FPNPs. (C) Fluorescence spectrum of FPNPs in the presence of different concentrations of MnCl_2_. (D) Stern-Volmer plot of the FPNPs at 0.1, 0.5, and 1 mg/mL in the presence of different concentrations of MnCl_2_. (E) Changes in the fluorescence intensity of MnCO_3_-FPNPs with the time and pH. (F) Fluorescence microscopic images of 4T1 cells after being treated with aqueous solutions of MnCO_3_-FPNPs at pH 7.4 and 5.4 (scale bar = 200 µm). Time-dependent flow cytometry histograms of 4T1 cells treated with (G) FPNPs and (H) MnCO_3_-FPNPs.

The FL quantum yield and the lifetime of the FPNPs were 12.5% and 2.96 ns, respectively (**Figure S9**). FPNPs have advantageous factors, such as an excellent pH stability and an excitation wavelength-dependent variation of the emission wavelength from blue to red (**Figure S10**). Interestingly, the FPNPs, which are structurally similar to PDA, have an ability to trap metal ions, such as Mn^2+^, Fe^2+^, Cu^2+^, and Ag^+^ species, through catechol-metal coordination and, consequently, the FL of the FPNPs is quenched via a photoinduced electron transfer process after metal chelation (**Figure S11**) 39-41. In this work, we chose Mn^2+^ as a chelating ion because it showed effective quenching efficiency for the FL of the FPNPs and since it can be used as a precursor ion for the mineralization of MnCO_3_ on the surface of the FPNPs. Therefore, the MnCO_3_-FPNPs, which were organized with the coordination of the FPNPs-Mn^2+^ complex, are able to controllably release Mn^2+^ through pH adjustment, indicating that the FL “OFF-ON” system can be implemented (**Figure 2A**). In neutral pH conditions, the original maximum peaks in the excitation and emission spectra of the FPNPs appear at 420 and 560 nm, respectively (**Figure 2B**). As the amount of added Mn^2+^ increases, the FL of the FPNPs gradually decreases, and when the concentration of Mn^2+^ reaches 200 mM, the initial FL of the FPNPs is quenched by 90% (**Figure 2C**). The FL quenching effect of Mn^2+^ on the FPNPs was estimated using the Stern-Volmer equation (equation (1)).

The FL quenching plots of Mn^2+^ were analyzed depending on the concentration of the FPNPs (**Figure 2D**). We observed that the FL of the FPNPs was effectively quenched in a broad FPNP concentration range (approximately 0.1-1 mg/mL) (**Figure 2D** and **Figure S12**). Notably, the coordination affinity in the catechol-metal complex can be weakened by decreasing the pH, which induces the protonation of the catechol hydroxyl groups 42. The MnCO_3_ exposed on the surface of the FPNPs is ionized to Mn^2+^ and CO_3_^2-^ under acidic conditions, thereby recovering the inherent FL through the FPNPs. In acidic conditions (pH 5.4), the FL intensity of the MnCO_3_-FPNPs increased dramatically from the quenched state and returned to the initial state of FL emission after 3 h, reflecting the release of Mn^2+^ ions from the FPNPs. Conversely, there was no significant change in FL intensity in physiological pH conditions (pH 7.4) (**Figure 2E**). **Figure S13** displays a visual representation of the dependency of the FL recovery of the MnCO_3_-FPNPs on the pH and time.

Next, we performed *in vitro* experiments using 4T1 cells to examine whether the MnCO_3_-FPNPs displayed their pH-activatable “OFF to ON” fluorescence property at the cellular level. To confirm the FL change of the MnCO_3_-FPNPs by cellular uptake, the MnCO_3_-FPNPs were dissolved in solutions of different pH (5.4 and 7.4), which were used to independently treat 4T1 cells for 3 h. Then, the nuclei were stained with the DAPI-mounting solution, and the fluorescence of the cells was observed. As a result, green FL was observed only in the cytoplasm of the 4T1 cells treated at pH 5.4, which is thought to be caused by the FL recovery from the MnCO_3_-FPNPs under acidic conditions (**Figure 2F**). In addition, the cellular uptake behavior of the FPNPs and MnCO_3_-FPNPs was further visualized. As shown in **Figure S14**, the FL signal from the FPNPs was observed in the cytoplasm of 4T1 cells after 2 h of treatment, whereas the FL signal from the MnCO_3_-FPNPs was observed after 3 h. To more accurately evaluate the cellular uptake of MnCO_3_-FPNPs, fluorescence intensity of 4T1 cells treated with FPNPs and MnCO_3_-FPNPs was measured by fluorescence-activated cell sorting (FACS) (**Figure 2G**-**H**). These results indicate that the FL from the MnCO_3_-FPNPs began to be recovered after the dissolution of MnCO_3_ within endo/lysosomal acidic compartments.

### pH-controlled MR performance

**Figure 3:**
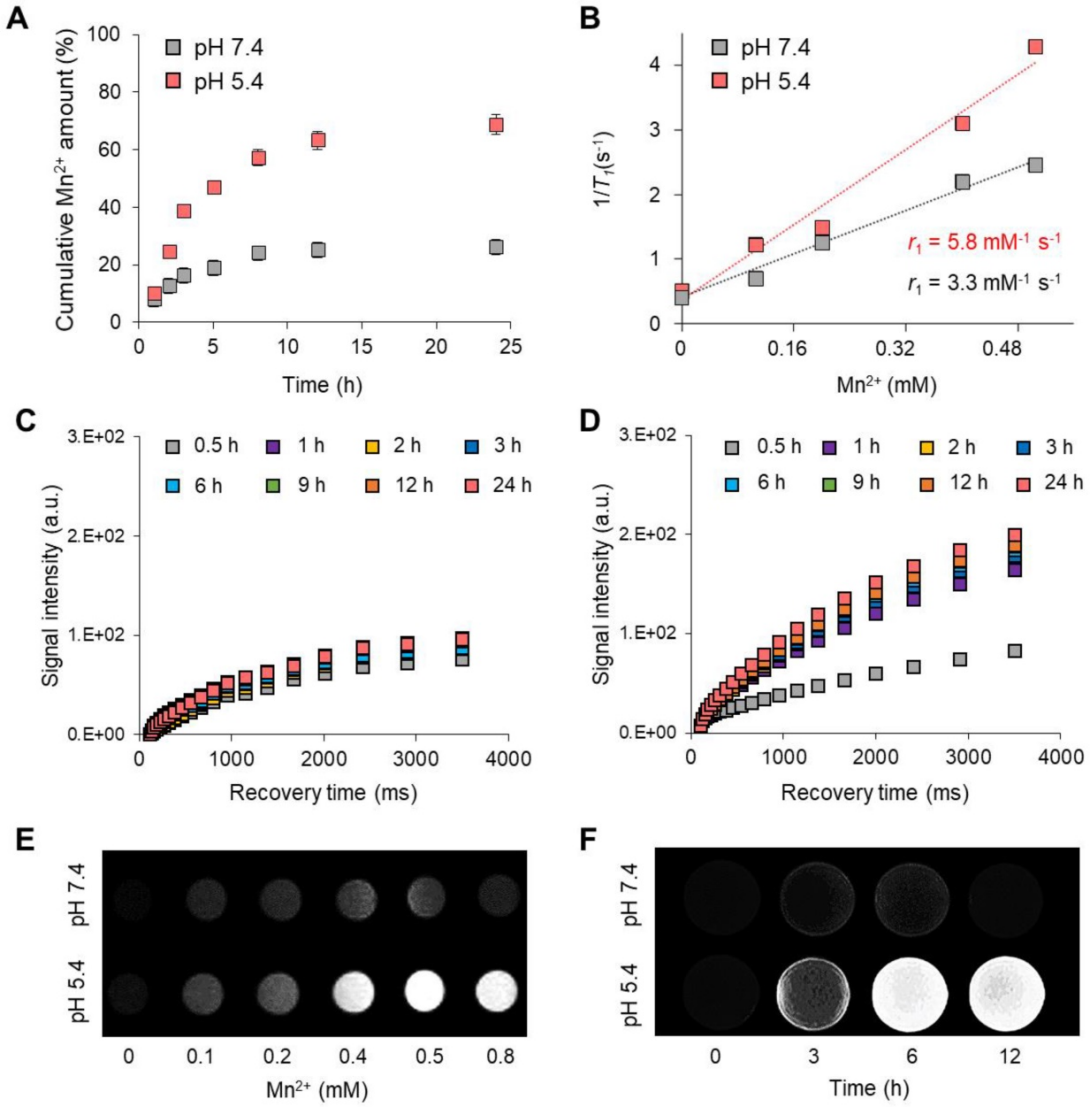
(A) Mn^2+^ release profiles of the MnCO_3_-FPNPs at pH 7.4 and 5.4. (B) Linear *T*_1_ relaxivity of the MnCO_3_-FPNPs at pH 7.4 and 5.4. Time-dependent longitudinal relaxation intensity of the MnCO_3_-FPNPs at (C) pH 7.4 and (D) pH 5.4. *T*_1_-Weighted phantom images of the MnCO_3_-FPNPs with (E) Mn^2+^ concentration and (F) time changes at pH 7.4 and 5.4.

Various manganese-based functional nanoparticles are used as *T*_1_-weighted contrast agents for cancer diagnosis 43, 44. In this study, the MnCO_3_ exposed on the surface of the FPNPs plays a vital role in the dual-modality imaging system because it can not only act as a *T*_1_-weighted contrast agent due to the MR-active Mn^2+^ releasing property, but also as an FL quencher of the FPNPs for the pH-activatable “OFF-ON” system. Such a pH-controlled dual-modality performance is extremely profitable to distinguish tumor cells from normal cells 45, 46. The Mn^2+^ released from MnCO_3_ provides *T*_1_-weight MR images, similar to gadolinium ions (Gd^3+^) 47, 48. **Figure 3A** shows the pH-controlled Mn^2+^ release behavior from the MnCO_3_-FPNPs. In acidic conditions (pH 5.4), the release of Mn^2+^ ions was accelerated, and the release amount of Mn^2+^ became higher (approximately two-fold) than that at pH 7.4. The Mn^2+^ release from the MnCO_3_-FPNPs was saturated after 9 h. The ability of the MnCO_3_-FPNPs to release Mn^2+^ in acidic conditions led to a strong *T*_1_ relaxation in MR. The relaxation rate *r*_1_ (1/* T*_1_) value of the MnCO_3_-FPNPs was 5.8 and 3.3 mM^-1^ s^-1^ at pH 5.4 and 7.4, respectively (**Figure 3B**). Meanwhile, there was no change in the transverse relaxation rate *r*_2_ (1/* T*_2_) values by varying the pH, which was determined to be 49.5 and 46.3 mM^-1^ s^-1^ for the same pH values, respectively (**Figure S15**). The relaxation rate *r*_1_ value of the MnCO_3_-FPNPs increased with a decreasing pH value, indicating that such MnCO_3_-FPNPs could serve as activatable *T*_1_-weighted magnetic resonance imaging (MRI) contrast agents. Compared with most Gd-chelated contrast agents (Gd-DOTA, 3.6 mM^-1^ s^-1^; and Gd-DTPA, 4.1 mM^-1^ s^-1^), the MnCO_3_-FPNPs at pH 5.4 induced a higher proton relaxivity 49.

The time-dependent longitudinal relaxation intensity of the MnCO_3_-FPNPs gradually increased, reaching a peak at 24 h after incubation at both pH 5.4 and 7.4 (**Figure 3C**-**D**). Taken together, the relaxivity of the MnCO_3_-FPNPs was found to be very sensitive to acidic pH. To verify the performance and effectiveness of the MnCO_3_-FPNPs as *T*_1_-contrast agents, the *T*_1_-weighted MR images of the MnCO_3_-FPNPs were captured at different concentrations and time at pH 7.4 and 5.4 (**Figure 3E**-**F**). As shown in the *T*_1_-weighted MR images, the brightness was clearly distinguished at a concentration higher than 0.4 mM in a pH 5.4 buffer and became gradually stronger after 3 h of incubation in acidic conditions.

### Photothermal conversion efficiency

**Figure 4:**
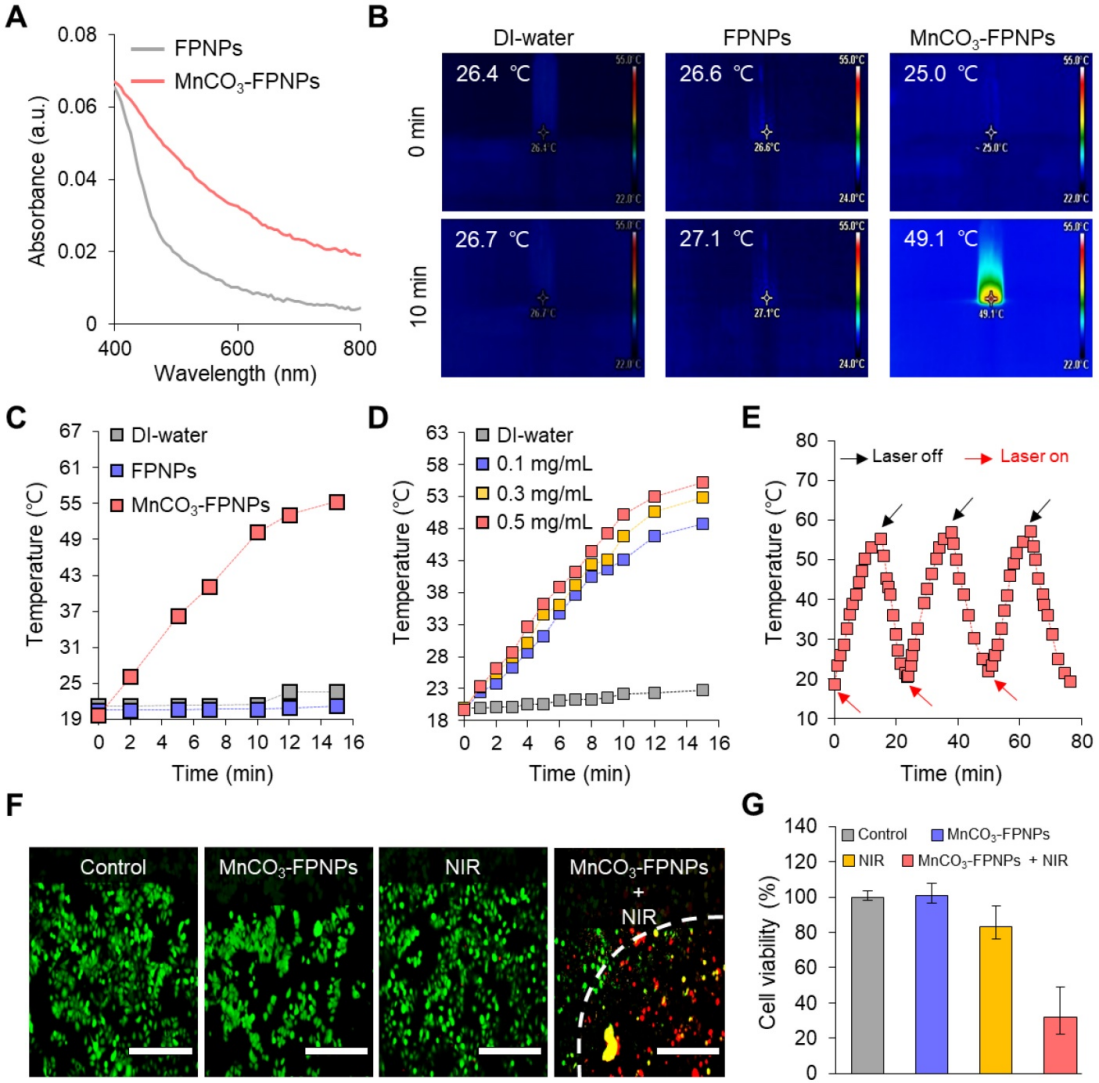
(A) Absorbance spectra of the FPNPs and MnCO_3_-FPNPs. (B) IR thermal images of DI water, aqueous solutions of FPNPs (0.5 mg/mL) and MnCO_3_-FPNPs (0.5 mg/mL) after continuous irradiation using a 808 nm laser (1.0 W cm^-2^) for 10 min, respectively. (C) Real-time temperature elevation of DI water, aqueous solutions of FPNPs (0.5 mg/mL), and MnCO_3_-FPNPs (0.5 mg/mL) under exposure to the 808 nm laser (1.0 W cm^-2^) for 15 min. (D) Photothermal conversion efficiency of DI water and aqueous solutions of MnCO_3_-FPNPs (0.1, 0.3, and 0.5 mg/mL) under exposure to the 808 nm laser (1.0 W cm^-2^) for 15 min. (E) Photothermal conversion stability of the MnCO_3_-FPNPs in three repetitions. (F) Fluorescence images of 4T1 cells stained using a live-dead kit belonging to the four groups (control, MnCO_3_-FPNPs, NIR, and MnCO_3_-FPNPs + NIR) and (G) their cell viability (*n* = 3) (scale bar = 400 µm).

We evaluated the *in vitro* photothermal therapeutic potential of the MnCO_3_-FPNPs by assessing their photothermal conversion efficiency. Importantly, we noted that the MnCO_3_-FPNPs showed a broad absorption from the visible to NIR region (400-900 nm), whereas the non-mineralized FPNPs showed a negligible absorption in the NIR range (Figure 4A). This indicates that the bandgap of the MnCO_3_-FPNPs was narrowed by the deposition of MnCO_3_ on the FPNP surface, which allowed the MnCO_3_-FPNPs to absorb NIR light and convert it into heat, thereby killing cancer cells 50. To further investigate the photothermal effect of MnCO_3_-FPNPs with a broad NIR absorption function, we recorded the heating and cooling curves of MnCO_3_-FPNPs solutions (0.5 mg/mL, 1.0 mL) through the process of turning the laser on and off (Figure S16A). Based on the linear fit of time/-ln(θ) obtained in the cooling process (Figure S16B) and Equation (3 and 4), the photothermal conversion efficiency (η) of MnCO_3_-FPNPs was calculated to be approximately 33%. To visualize the photothermal conversion efficiency of the MnCO_3_-FPNPs, the temperature change of DI water and aqueous solutions of FPNPs (0.5 mg/mL) and MnCO_3_-FPNPs (0.5 mg/mL) was independently measured using an infrared (IR) thermal imaging camera under continuous NIR laser irradiation (808 nm) for 10 min (Figure 4B). After 10 min of NIR irradiation (1.0 W cm^-2^), a temperature increase from 25.0 ℃ to 49.1 ℃ was only observed in the MnCO_3_-FPNP solutions. More detailed temperature variations of each solution were recorded live for 15 min (Figure 4C).

Taken together, the MnCO_3_-FPNPs not only have excellent photothermal conversion efficiency, but also exhibit photothermal killing performance *in vitro* under NIR laser irradiation. This indicates that the photothermal conversion occurs due to the broad absorbance of the MnCO_3_-FPNPs in the NIR region. For equal laser power (1.0 W cm^2^), the photothermal conversion efficiency of the MnCO_3_-FPNPs increased with increasing concentration (**Figure 4D**). The photothermal stability of the MnCO_3_-FPNPs showed prominent photostability without deterioration in 3 laser “ON-OFF” cycles (**Figure 4E**). The good water solubility and excellent biocompatibility of the MnCO_3_-FPNPs allowed the estimation of the *in vitro* photothermal therapeutic activity via NIR irradiation. Based on cytotoxicity evaluation (**Figure S17**), we found that the MnCO_3_-FPNPs uptake into normal cells and cancer cells exhibited low cell toxicity up to a concentration of 500 µg/mL. To induce cell death by hyperthermia, 0.5 µg/mL MnCO_3_-FPNPs were used to treat 4T1 cells. After 12 h of treatment, fluorescent cellular live-dead staining images were obtained for the four sample groups. During the exposure to an 808 nm laser (1.0 W cm^-2^) for 10 min, the 4T1 cells died due to hyperthermia, which was confirmed by the red fluorescence in the MnCO_3_-FPNPs + NIR group (**Figure 4F**), and the death of 4T1 cells tended to increase as the laser power increases (**Figure S18**). On the contrary, 4T1 cells exhibited green fluorescence in the control (PBS), MnCO_3_-FPNPs, and NIR groups, which suggests that the cells survived, and no cell death was induced by hyperthermia in those groups (**Figure 4F**). In this regard, the cell viability in the MnCO_3_-FPNPs + NIR group was less than 40%, while that in the other groups was higher than 80% (**Figure 4G**).

### *In vivo* activatable dual-modality imaging

**Figure 5:**
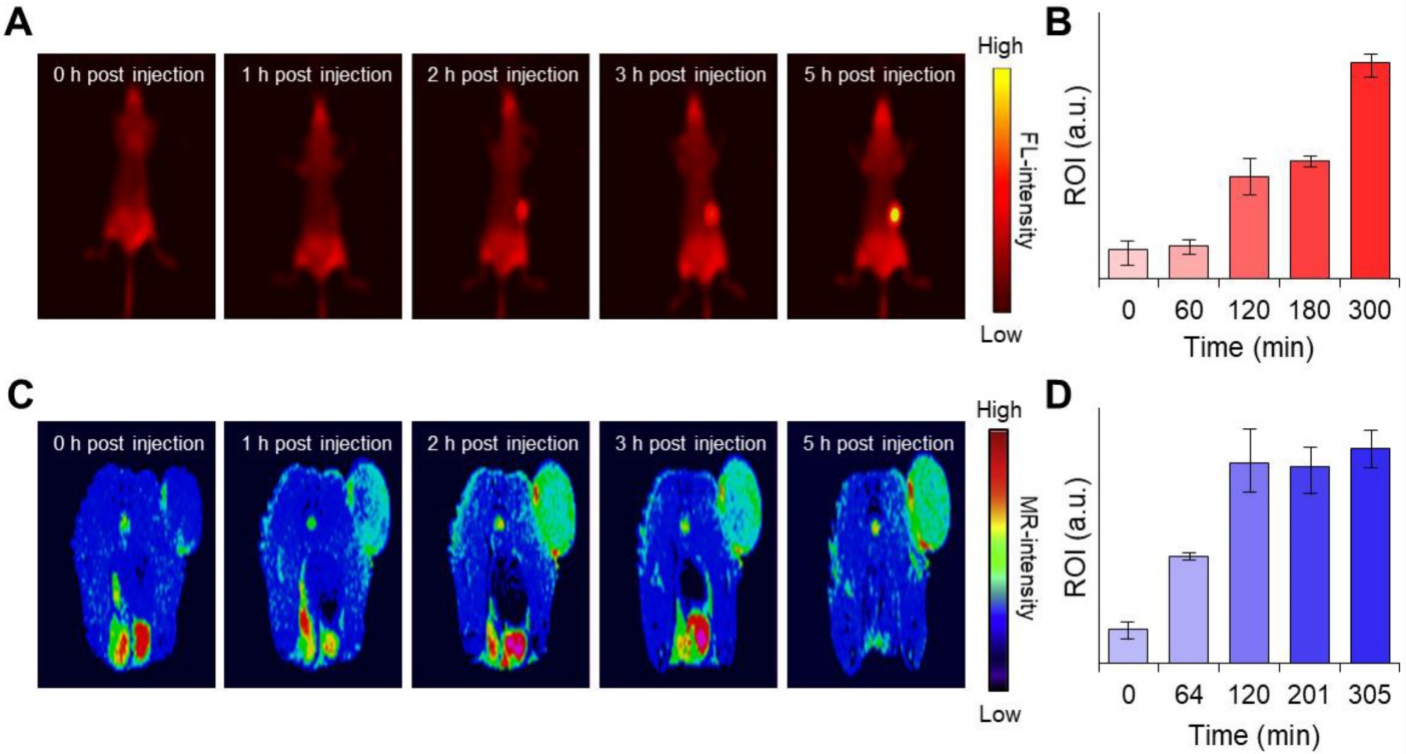
(A) Time-dependent fluorescence images of the 4T1 tumor-bearing balb/c nude mice after intravenous injection of MnCO_3_-FPNPs using Cy 3.5 filter and (B) their fluorescence intensity in the tumor site (*n* = 3). (C) Time-dependent *T*_1_-weighted MR images of 4T1 tumor-bearing balb/c nude mice after intravenous injection of MnCO_3_-FPNPs and (D) their MR intensity in the tumor site (*n* = 3).

Next, we were interested in knowing whether the MnCO_3_-FPNPs exhibit an activatable “OFF-ON” performance for the FL and we performed *T*_1_-MR imaging of an *in vivo* tumor model. Interestingly, synthesized FPNPs have excitation-dependent emission wavelengths similar to carbon dots, enabling FL imaging *in vivo* (Figure S19) 51, 52. For the time-dependent *in vivo* FL imaging, the MnCO_3_-FPNPs dissolved in PBS were administered intravenously into 4T1 tumor-bearing balb/c nude mice (tumor volume =100 mm^3^). Three hours post-injection of the MnCO_3_-FPNPs, a remarkable FL signal without the non-specific signal was shown at the tumor site, suggesting an activatable “OFF-ON” imaging ability, which enables the visualization of an effective tumoral accumulation of the MnCO_3_-FPNPs (Figure 5A-B). In contrast, the FL signal of 4T1 tumor-bearing mice, which were injected with the non-mineralized FPNPs, showed a strong non-specific signal, predominantly in the liver and in the tumor from 1 to 3 h post-injection (Figure S20). In a short period (within 3 h), the FL intensity of the MnCO_3_-FPNPs at the tumor site seemed rather lower than that of the FPNPs because the emission of FL from the MnCO_3_-FPNPs was accelerated with excellent photostability after the dissolution of MnCO_3_ minerals within the acidic endosomes (pH ~6.5) and/or lysosome (pH ~4.5) in cancer cells (Figure S21). In order to specifically evaluate the tumor accumulation of the MnCO_3_-FPNPs, *ex vivo* FL images were obtained for the heart, lungs, liver, spleen, kidneys, and tumors 24 h after the intravenously injection of PBS, FPNPs (0.5 mg/mL), and MnCO_3_-FPNPs (0.5 mg/mL) into 4T1 tumor-bearing mice (Figure S22). We found that the MnCO_3_-FPNPs showed pronounced FL at the tumors but showed weak signals in the other organs, such as the liver and kidneys. The higher FL of the MnCO_3_-FPNPs than the FPNPs at the tumor is probably ascribed to the effect of the surface MnCO_3_. MnCO_3_ deposited on the FPNPs surfaces may improve the structural stability of the MnCO_3_-FPNPs during blood circulation, thereby resulting in effective activation of strong FL imaging at tumor tissues.

We investigated whether surface mineralization can improve the stability of the MnCO_3_-FPNPs in the serum condition (pH 7.4). This serum stability was compared with that of the simple metal-bound Mn-FPNP (a non-mineralized control). The changes in fluorescence intensities of the MnCO_3_-FPNPs and the Mn-FPNPs in the serum solution were observed. As shown in **Figure S23**, the MnCO_3_-FPNPs maintained the initial intensity of quenched fluorescence in the PBS and also even in the FBS-containing PBS solution. This behavior indicates that the robust structure of MnCO_3_ minerals may keep the Mn-catechol complex state stable in the serum condition. In contrast, the fluorescence intensity of the Mn-FPNPs gradually increased in FBS-containing PBS solution, which resulted in non-specific fluorescence signals *in vivo*. It is likely that the serum proteins readily interact with the surface-exposed chelated Mn^2+^ ions of the Mn-FPNPs and thus may weaken the coordination force between Mn^2+^ and catechol groups, thereby resulting in the recovery of FPNPs fluorescence. These results indicate that the existence of surface MnCO_3_ on the FPNPs is effectively resistant to the interaction with serum proteins, whereas the Mn-FPNPs may be interfered with various serum proteins.

Hence, it is obvious that MnCO_3_ mineralization on the FPNPs could offer the benefits not only in tumor specificity of FL activation but also in nanoparticle stability, compared with the simple ionic Mn^2+^ chelation on the FPNPs. Likewise, a *T*_1_-weighted “OFF-ON” MR signal was also observed at a similar time in the tumors of the 4T1 tumor-bearing mice injected with the MnCO_3_-FPNPs, and the signal was saturated after 2 h (**Figure 5C**-**D**). The *T*_1_-weighted MR signal was not detected in the FPNPs-treated mice until 3 h post-injection (**Figure S24**). These results indicate that in the acidic endosomes of cancer cells, the MnCO_3_-FPNPs efficiently released Mn^2+^ ions, thereby resulting in a *T*_1_-weighted MR contrast enhancement.

### *In vivo* PTT

**Figure 6:**
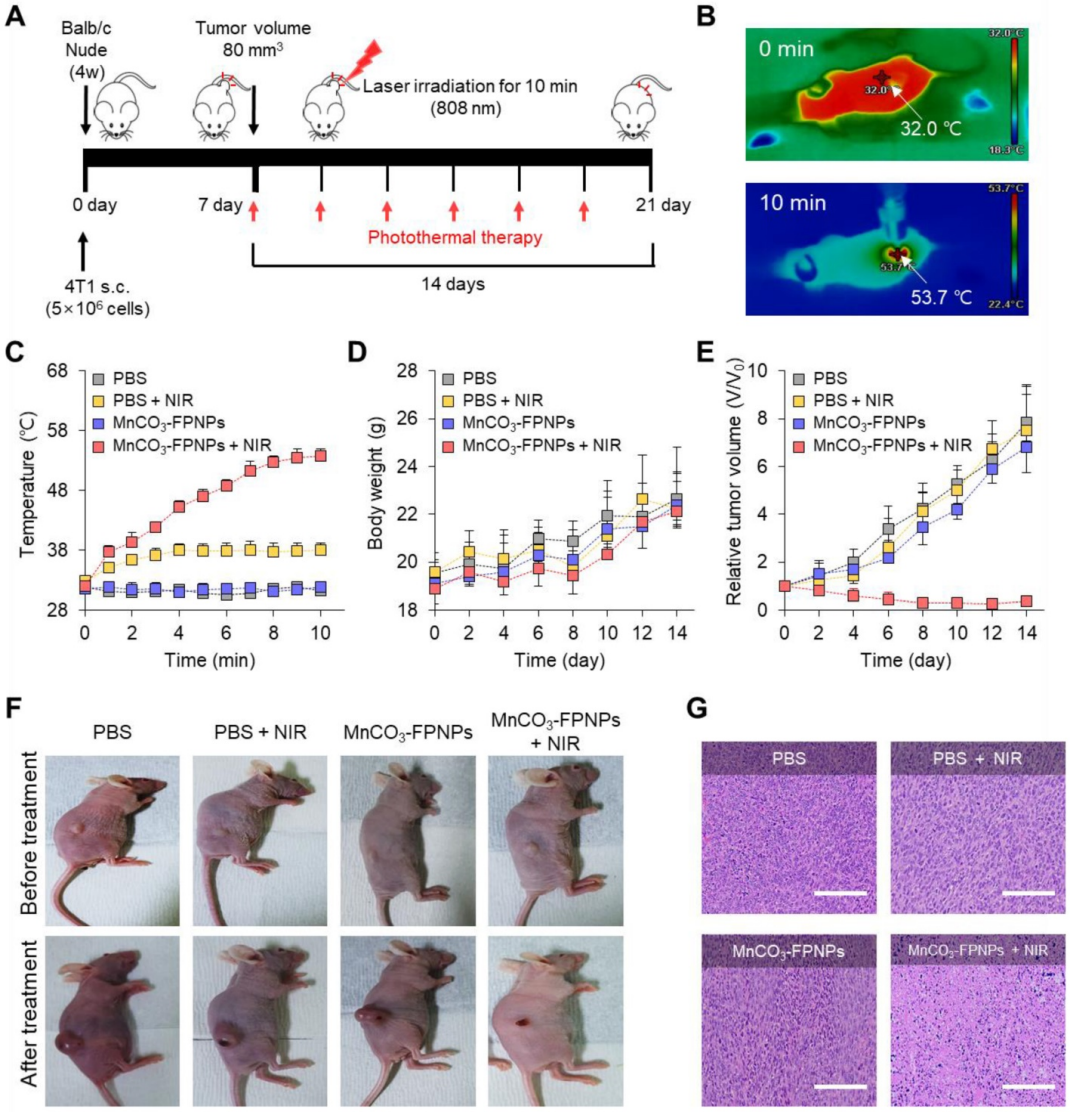
(A) Schematic diagram of the photothermal treatment period and processing method of MnCO_3_-FPNPs *in vivo*. (B) IR thermal camera images of balb/c nude mice injected with MnCO_3_-FPNPs under continuous NIR-laser irradiation for 10 min. (C) *In vivo* photothermal conversion efficiency of PBS and MnCO_3_-FPNPs intravenous injected 4T1 tumor-bearing mice for 10 min under continuous irradiation with an 808 nm laser. (D) Monitoring of the average body weight and (E) relative tumor volume during therapy for 14 days. (F) Camera photographs of the mice before and after PTT. (G) Hematoxylin and eosin (HE) staining images of the tumor tissue after PTT (scale bar = 200 µm).

To further evaluate the photothermal therapy effect of the MnCO_3_-FPNPs irradiated at 808 nm in the 4T1 tumor-bearing mice, the detailed treatment method is presented in **Figure 6A.** To monitor the *in vivo* temperature variation, PBS and the MnCO_3_-FPNPs (0.5 mg/mL) were independently administered to 4T1 tumor-bearing balb/c nude mice (5-weeks old) via an intravenous injection. The temperature variation in the tumor sites under 808 nm laser (1.0 W cm^-2^) irradiation was recorded using an IR thermal camera during the PTT process. It is noteworthy that the temperature of the tumor sites in the mice injected with the MnCO_3_-FPNPs increased from 32 ℃ to 53.7 ℃ within 10 min (**Figure 6B**-**C**). In contrast, the temperature in the mice injected with PBS did not change significantly in the same exposure conditions. In this regard, the IR thermal images of the 4T1 tumor-bearing mice depending on the laser power and time variation are presented in **Figure S25** and **S26**. During the PTT process, the changes in the body weight and tumor volume were measured in the 4T1 tumor-bearing balb/c nude mice every 2 days for 14 days (**Figure 6D**-**E**). For 14 days, there was no remarkable change in the body weight in the mice of all groups (PBS, PBS + NIR, MnCO_3_-FPNPs, and MnCO_3_-FPNPs + NIR).

On the other hand, the volume of the tumors was remarkably reduced for the mice in the MnCO_3_-FPNPs + NIR group, indicating an effective ablation of tumor cells and that the MnCO_3_-FPNPs worked well as a PTT agent (**Figure 6E**-**F**). The reduction in the tumor volume by PTT is visually represented in **Figure S27**, which was obtained by dissecting the tumors. To not only discover the major hyperthermia mechanism involved in killing cancer cells via stimulating tumor shrinkage, but also the potential toxicity of the MnCO_3_-FPNPs in mice, histological analysis using hematoxylin and eosin (HE) staining was performed in the mice of all groups and in the major organs (**Figure S28**). Histologically, the MnCO_3_-FPNPs with NIR showed more disruption, nuclear shrinkage, and necrosis in the tumor tissues via damage induced by photothermal ablation than the agents used in the other groups (**Figure 6G**). These histological evaluation results suggest that the MnCO_3_-FPNPs can serve as a dual-imaging-guided PTT agent, having low biological toxicity. FL/MR imaging-guided PTT of tumors after tumoral accumulation by the enhanced permeability and retention (EPR) effect.

## Conclusions

We developed a novel activatable dual-modality imaging-guided PTT theranostic agent based on MnCO_3_-mineralized polydopamine nanoparticles (MnCO_3_-FPNPs). The MnCO_3_-FPNPs were successfully fabricated via the well-controlled deposition of crystalline MnCO_3_ minerals on the surface of the FPNPs. The MnCO_3_-FPNPs had a unique FL/MR dual-modality “OFF-ON” imaging ability in response to pH variation. The MnCO_3_-FPNPs exhibited a potential diagnostic ability, as observed through activatable FL/MR imaging of the 4T1 tumor-bearing mice. In addition, the cancer treatment efficiency of the MnCO_3_-FPNPs using a NIR laser could be accomplished in 4T1 tumor-bearing mice within 14 days. We verified that the MnCO_3_ mineralization approach on the surface of FPNPs was beneficial for designing novel theranostic nanoparticles, which exhibited unique “OFF-ON” activation abilities in FL/MR dual imaging and PTT functions. Our proof-of-concept studies suggest that the MnCO_3_-FPNPs may serve as a useful platform for various mineralized PDA-based multimodal imaging-guided PTT models for many cancer theranostic applications.

## Materials and Methods

### Materials and instruments

Dopamine hydrochloride (DA HCl), EDA (99.5% purity), manganese chloride (MnCl_2_), sodium carbonate (Na_2_CO_3_), ammonium hydroxide solution (28%), ethanol, 4′,6-diamidino-2-phenylindole (DAPI), a live/dead cell double staining kit, and a CCK-8 kit were purchased from Sigma Aldrich (St. Louis, MO, USA). Roswell Park Memorial Institute (RPMI) 1640 medium, fetal bovine serum (FBS), and penicillin-streptomycin were purchased from Thermo-Fisher (Waltham, MA, USA). 4T1 cells were obtained from the American Type Culture Collection (ATCC, CRL-2539). All reagents were used without any dilution or desalting. An UV-vis spectroscopy (Beckman Coulter, DU 800), a fluorescence spectrometer (Scinco FluoroMate FS-2, Seoul, South Korea), a time-correlated single photon-counting system (Horiba Ltd., Kyoto, Japan), and a luminescent image analyzer (LAS-4000, Fujifilm, Tokyo, Japan) were used for optical analysis. Dynamic light scattering (DLS) and zeta potential measurements were performed using a particle size analyzer (Malvern Zetasizer Nano ZS, UK). Structural characterization was performed using Fourier-transform infrared spectroscopy (FT-IR, Bruker Corp., Billerica, MA, USA), powder XRD (Rigaku-Ultima IV), high resolution transmission electron microscopy (HR-TEM, G2 F30X-TWIN, 300 kV), scanning electron microscopy (SEM, JEOL microscope), EDS (JEOL microscope), and X-ray photoelectron spectroscopy (XPS, Thermo-Fisher Waltham, MA, USA). Inductively coupled plasma-atomic emission spectroscopy (ICP-AES) (OPTIMA 7300 DV, Perkin-Elmer, USA) was used to create the Mn^2+^ release profiles. *In vitro* cellular assays were performed using confocal microscopy (Olympus FV 1000, Olympus Corp, Tokyo, Japan), cytometry sorter system (FACS, Becton Dickinson, New York, USA) a microplate reader (SpectraMax M2/Molecular Devices), and an EVOS FL Cell Imaging System (Thermo-Fisher, Waltham, MA, USA). *In vivo* fluorescence images were obtained using an IVIS Lumina *in vivo* imaging system (Caliper LifeSciences, USA). All MR experiments were performed using a 4.7 T animal magnetic resonance imaging (MRI) scanner (BioSpec 47/40, Bruker, Germany) at Korea Basic Science Institute (Ochang, Republic of Korea).

### Synthesis of FPNPs

The FPNPs were synthesized from DA HCl and EDA in a mixed weak alkaline solution composed of an aqueous ammonia solution (5 mL), ethanol (80 mL), and deionized (DI) water (180 mL). The detailed procedure used for the preparation of the FPNPs was as follows: firstly, DA HCl (100 mg) was dissolved in a mixture of EDA (3 mL) and a weak alkaline aqueous solution (pH 8.5, 30 mL). The polymerization required to prepare the FPNPs was allowed to proceed for 12 h under continuous stirring condition. Afterward, the resulting light-brown solution was washed by using a centrifugal filter (30 kDa) and dialyzed using a dialysis membrane (MWCO: 10 kDa) in DI water for 2 days to remove the impurities. Finally, the purified solution was lyophilized for 2 days to obtain the FPNPs. As a control nanoparticle, the PDA nanoparticle was synthesized from DA HCl without EDA.

### MnCO_3_ mineralization of the FPNPs

The FPNPs (10 mg) were dissolved in DI water (10 mL). Subsequently, an aqueous MnCl_2_ solution (200 mM) was added to the aqueous FPNP solution, followed by stirring at room temperature for 12 h. The resulting opaque solution was purified using a centrifugal filter (30 kDa) to remove the unreacted ions. Then, an aqueous solution of Na_2_CO_3_ (200 mM) was added to the purified FPNP solution, in which the manganese ions (Mn^2+^) were chelated on the catecholic OH groups of the FPNP surfaces. After vigorous stirring at room temperature for 12 h, the resulting mineralized MnCO_3_-FPNPs were isolated via centrifugation and washed with DI water three times, followed by lyophilization. Fluorescence Quenching Analysis: The FPNPs (10 mg) were dissolved in DI water (10 mL). The Mn^2+^-dependent FL quenching relationship among the FPNPs was estimated using the Stern-Volmer equation (1).

*F_0_*/*F* = 1 + *K*_SV_ [Q] (1)

Where *F_0_* and *F* are the FL intensities of the FPNPs measured at 550 nm in the absence and presence of Mn^2+^ ions, respectively. *K*_SV_ is the Stern-Volmer quenching constant and [Q] is the concentration of the quencher (Mn^2+^).

### Theoretical calculations

The energy levels of the molecular orbitals of PDA and FPNPs were calculated through DFT by Gaussian 16 program. The molecular orbitals of possible moieties were imported at the B3LYP/6-31 G(d, p) level.

### Calculation of the fluorescence quantum yield

The FL quantum yield (Q) of the FPNPs was evaluated using a previously reported method 53, 54. Rhodamine 6G (Q = 95%) dispersed in ethanol was employed as a standard fluorophore. The FL quantum yield of the FPNPs was calculated according to the equation (2).

Q = 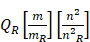

(2)

Where m, n, and the subscript *R* represent the slope of the line obtained from the plot of the integrated fluorescence intensity vs. absorbance, the refractive index of the solvent, and the reference fluorophore of the known quantum yield, respectively. pH-Controlled Release of the Manganese Ions: To demonstrate the accelerated release of Mn^2+^ ions from the MnCO_3_-FPNPs in acidic conditions, the MnCO_3_-FPNPs solutions were dialyzed using a dialysis membrane bag (MWCO: 500-1,000 Da) and a 0.1 M PBS buffer (pH 7.4) or a 0.1 M acetate buffer (pH 5.4). In order to estimate the release behavior of Mn^2+^ ions from the MnCO_3_-FPNPs with time at endosomal acidic pH, the dialyzed solution was analyzed using ICP-AES.

### pH-controlled MR relaxivity

To quantitatively evaluate the pH-dependent *T*_1_ and *T*_2_, the MnCO_3_-FPNPs (0.8 mM) were prepared in 0.1 × PBS buffer (pH 7.4) and 0.1 M acetate buffer (pH 5.4), respectively. The *T*_1_ and *T*_2_ relaxation time, and the corresponding *T*_1_ and *T*_2_-weighted images of the MnCO_3_-FPNPs were obtained using a 4.7 T animal MRI scanner with 72 mm volume coils for radiofrequency transmission and reception.

### Visualization of the cellular uptake and cytotoxicity assay

The monitoring of the cellular uptake and a cytotoxicity assay for the control FPNPs and the MnCO_3_-FPNPs were performed using 4T1 breast cancer cells. To visualize the cellular uptake of the FPNPs and the MnCO_3_-FPNPs, 4T1 cells were cultured in α-MEM buffer with 10% FBS and 1% penicillin-streptomycin in a humidified incubator under 5% CO_2_ at 37 ℃. The initial density of the cultured 4T1 cells was 1 × 10^5^ cells/100-mm cell dish, and the medium was changed every 3 days. For the cytotoxicity assay, a CCK-8 kit was used. Briefly, each 100 µL of the CCK-8 solution was added to the FPNPs-, Mn-FPNPs-, and MnCO_3_-FPNPs-treated 4T1 cells on 96-well plates. After 30 min of incubation, the treated cells were washed with PBS and the absorbance was measured at 450 nm using a microplate reader. All the experiments were performed in triplicate, and the relative cell viability (%) was expressed as a percentage relative to that of the untreated control cells.

### Flow cytometry analysis

To examine the cellular uptake, 4T1 cells cultured in 96-well plate at a concentration of 1 × 10^4^ cells/well were treated with FPNPs and MnCO_3_-FPNPs for 2, 3, 6, and 12 h. After incubation, treated 4T1 cells washed twice with fresh medium and collected in the dark. Subsequently, the fluorescence intensity of the collected cells was measured by a cytometry sorter system with laser excitation at 488 nm.

#### *In vitro* photothermal conversion efficiency

Photothermal conversion efficiency (η) of the MnCO_3_-FPNPs was calculated using the following equation (3) by the previous reported study 55.


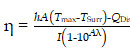

(3)

where *h* is the heat transfer coefficient, *A* is the surface area of the container, *T*_max_ is the equilibrium temperature, *T*_Surr_ is the ambient temperature of the environment, *Q*_Dis_ expresses the heat dissipation from the light absorbed by the quartz sample cell, *I* is an incident laser power, and *A*λ is the absorbance of MnCO_3_-FPNPs at 808 nm. The value of *hA* was calculated using the following equation (4).


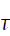
_s_ = 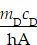

(4)

where *τ*_s_ is the sample system time constant; *m*_D_ and *c*_D_ are the mass and heat capacity of the deionized water used as solvent, respectively.

To explore the real-time temperature variation of the MnCO_3_-FPNPs, aqueous MnCO_3_-FPNPs solutions at various concentrations (from 0.1 mg/mL to 1 mg/mL) were irradiated with NIR light. In brief, the aqueous MnCO_3_-FPNPs solutions were irradiated for 15 min using an 808 nm laser (2 W cm^-2^). The photothermal conversion stability of the MnCO_3_-FPNPs was measured under three cycles of an “ON-OFF” control of 15 min using an 808 nm laser (2 W cm^-2^). All the experiments used to determine the photothermal conversion efficiency were recorded using an IR thermal camera.

### *In vitro* PTT efficacy

For the cellular PTT assay, 4T1 cells were cultured on 96-well plates at a density of 5 × 10^3^ cells well^-1^ and incubated for 1 day in a humidified incubator under at 5% CO_2_ and 37 ℃. Then, the cultured 4T1 cells were treated with the MnCO_3_-FPNPs for 12 h. Finally, the treated cells were washed with PBS three times and irradiated with an 808 nm laser (1.0 W cm^-2^) for 10 min. The cell viability of the irradiated cells and untreated control cells was assessed using a CCK-8 kit and a live-dead cell staining kit according to the manufacturers' instructions.

### Tumor animal model

To establish the breast tumor balb/c nude mouse model, approximately 5 × 10^6^ 4T1 cells dispersed in 100 μL of serum-free α-MEM were subcutaneously injected into the 5-week-old female balb/c nude mice. When the tumor volume reached approximately 80 mm^3^, the 4T1 tumor-bearing mice were randomly separated into four different groups (4 mice in each group) for the *in vivo* experiments: i) PBS, ii) PBS + NIR, iii) MnCO_3_-FPNPs, and iv) MnCO_3_-FPNPs + NIR. The balb/c nude mice used for *in vivo* imaging and the PTT experiments were anesthetized using isoflurane and a gas anesthesia system. All experimental animal care and handling procedures were carried out in accordance with the guidelines from the Korea Research Institute of Bioscience and Biotechnology (KRIBB), and all of experimental protocols were approved by KRIBB-IACUC (approval number: KRIBB-AEC-20152).

### *In vivo* dual-modality imaging

*In vivo* FL imaging of the 4T1 tumor-bearing balb/c nude mice was performed using an IVIS imaging system using Cy 3.5 filter following a tail vein injection of 100 μL of the MnCO_3_-FPNPs and the FPNPs, respectively. *In vivo T*_1_-weighted MR imaging of the 4T1 tumor-bearing balb/c nude mice was performed at predetermined time intervals after the tail vein injection of 100 μL of the MnCO_3_-FPNPs (0.5 mg/mL) using a 4.7 T animal MRI scanner with the following parameters: a field of view of 50 × 50 mm, a 128 × 128 matrix, a repetition time (TR) of 281.2 ms, an echo time (TE) of 7.5 ms, and a slice thickness of 1 mm.

### *In vivo* PTT

For evaluating the photothermal therapeutic effects, the 4T1 tumor-bearing mice were treated via intravenous injection with 100 µL of the MnCO_3_-FPNPs (0.5 mg/mL) in a PBS solution. Subsequently, the MnCO_3_-FPNP-injected mice were continuously irradiated with an 808 nm laser of a power density of 1.5 W cm^-2^ for 10 min (*n* = 4). After irradiation, the size of the tumors of the mice was measured using a caliper every 2 days for 14 days, and the tumor volume was calculated using the following equation (5).


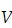
 = 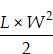

(5)

Where *V* is the calculated tumor volume, L is the length of the tumor, and *W* is the width of the tumor.

### Histological analysis

Histological analysis (HE staining) of the extracted organs was performed as follows: first, the extracted organs were fixed in neutral buffered formalin and were sliced into slices with a thickness of approximately 2-3 mm and with a shape suitable for tissue specimen production. Then, the specimens that had been trimmed appropriately for the production of the tissue specimens were placed in a cassette with an individual number and subjected to tissue treatment for 13 h. Next, the treated tissues were cut into slices with a thickness of approximately 3-4 µm using a microtome, were attached to a slide, and dried. Afterward, the dried specimens were washed with distilled water after deparaffinization and rehydration. Finally, HE staining was carried out using the standard method.

## Supplementary Material

Supplementary figures.

## Abbreviations

MnCO_3_-FPNPs: MnCO_3_-mineralized fluorescent polydopamine nanoparticles
FPNPs: fluorescent PDA nanoparticles
PTT: photothermal therapy
MR: magnetic resonance
FL: fluorescence
PET: positron emission tomography
CT: computed tomography
US: ultrasound
NIR: near-infrared
SPIONs: superparamagnetic iron oxide nanoparticles
Gd: gadolinium
PDA: polydopamine
EDA: ethylenediamine
TEM: transmission electron microscopy
HR-TEM: high resolution transmission electron microscopy
SEM: scanning electron microscopy
EDS: energy-dispersive X-ray spectroscopy
XRD: Powder X-ray diffraction
XPS: X-ray photoelectron spectroscopy
Gd-DOTA: gadolinium-tetraazacyclo dodecanetetraacetic acid
Gd-DTPA: gadolinium-diethylenetriaminepentaacetic acid
HE: hematoxylin and eosin
EPR: enhanced permeability and retention
DLS: dynamic light scattering
FT-IR: Fourier-transform infrared spectroscopy
ICP-AES: inductively coupled plasma-atomic emission spectroscopy
