# Supplementary material for: MnCO3-mineralized polydopamine nanoparticles as an activatable theranostic agent for dual-modality imaging-guided photothermal therapy of cancers
Source: Theranostics. 2022 Sep 21;12(15):6762–78. doi: 10.7150/thno.77060 (PMC9516237; doi:10.7150/thno.77060)
Supplement: Supplementary file 1 — Supplementary figures. [file thnov12p6762s1.pdf]

Supplementary Material

$MnCO_3$-mineralized polydopamine nanoparticles as an activatable theranostic agent for dual-modality imaging-guided photothermal therapy of cancers

Kyung Kwan Lee[1,2], Jae-Hyung Lee[3], Sang Cheon Lee[4,*] and Chang-Soo Lee[1,5,*]

1. Bionanotechnology Research Center, Korea Research Institute of Bioscience and Biotechnology (KRIBB), Daejeon 34141, Republic of Korea
2. Department of Life and Nanopharmaceutical Sciences, Graduate School, Kyung Hee University, Seoul 02447, Republic of Korea
3. Department of Oral Microbiology, School of Dentistry, Kyung Hee University, Seoul 02447, Republic of Korea
4. Department of Maxillofacial Biomedical Engineering, School of Dentistry, Kyung Hee University, Seoul 02447, Republic of Korea
5. Department of Biotechnology, University of Science & Technology (UST), Daejeon 34113, Republic of Korea

*Corresponding authors: Prof. Sang Cheon Lee, email: schlee@khu.re.kr; Dr. Chang-Soo Lee, email: cslee@kribb.re.kr

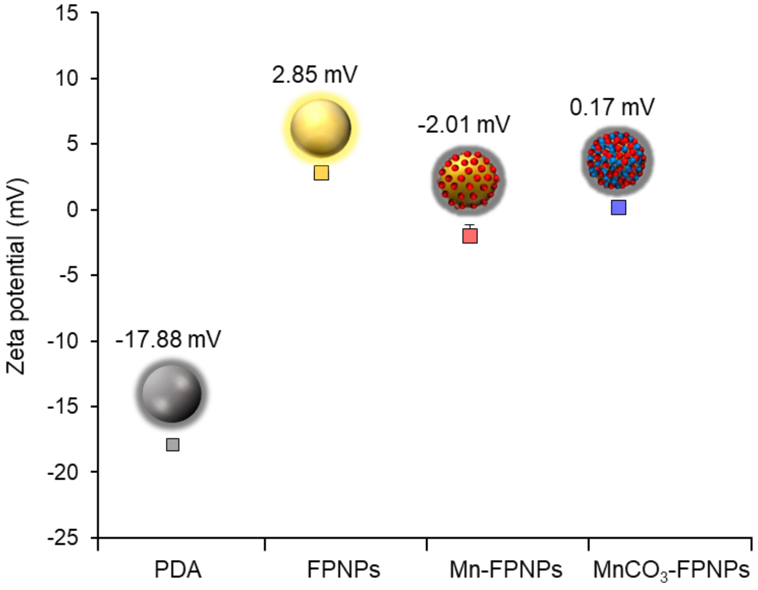

Figure S1. Zeta-potential values of control PDA nanoparticles, FPNPs, Mn-FPNPs, and $MnCO_3$-FPNPs in deionized water.

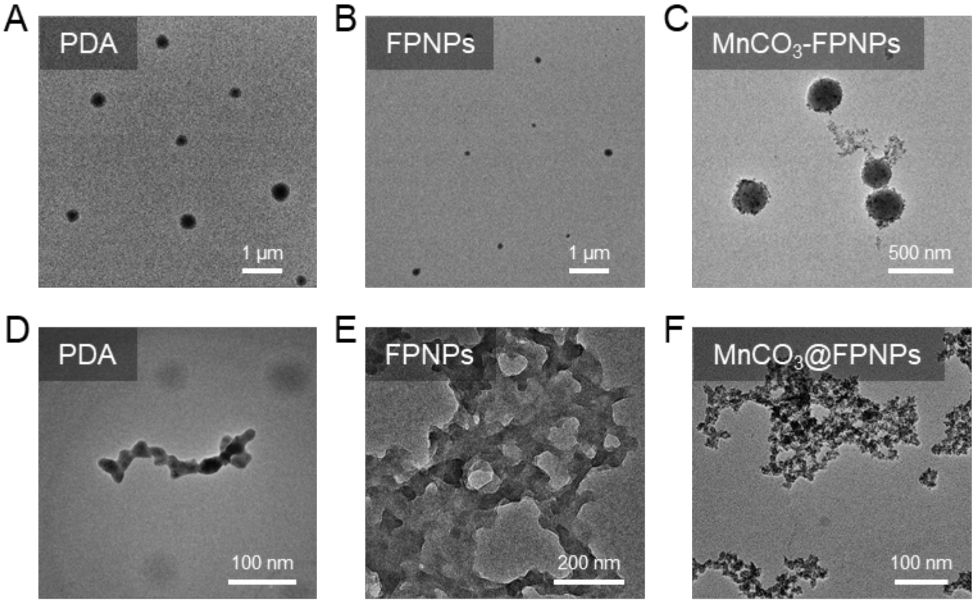

Figure S2. TEM images of (A) control PDA nanoparticles, (B) FPNPs, and (C) $MnCO_3$-FPNPs prepared from ammonia solution. TEM images of (D) control PDA nanoparticles, (E) FPNPs, and (F) MnCO3-FPNPs prepared from Tris-HCl.

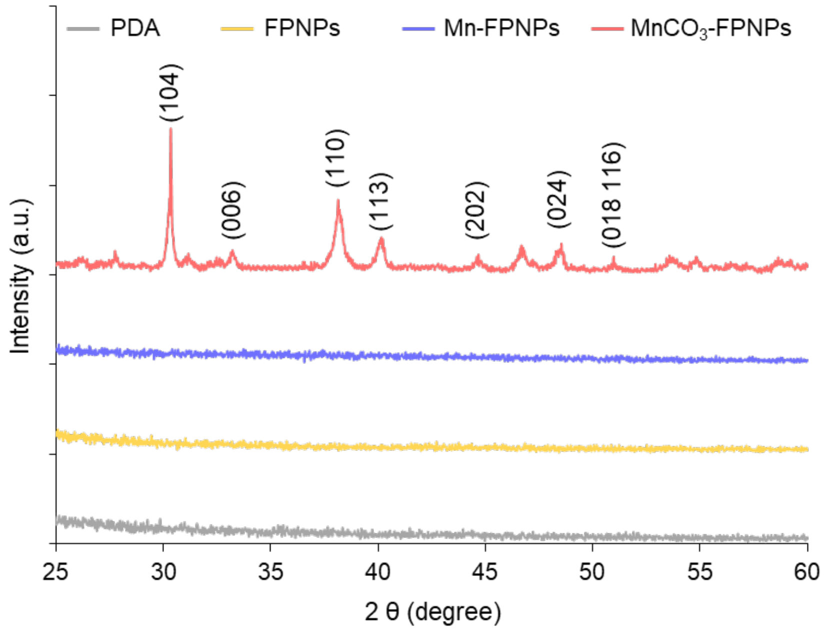

Figure S3. The powder XRD patterns of control PDA nanoparticles, FPNPs, Mn-FPNPs and $MnCO_3$-FPNPs.

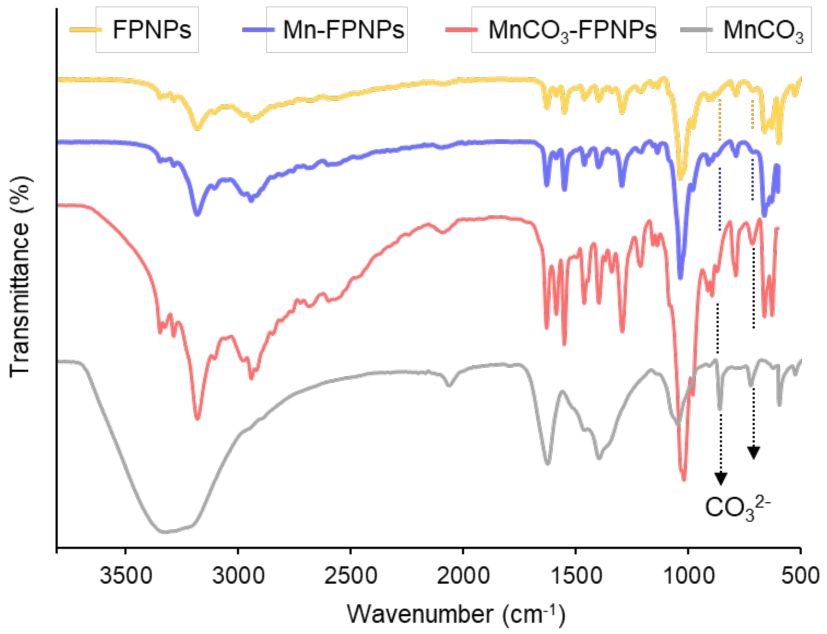

Figure S4. FT-IR spectra of FPNPs, Mn-FPNPs, $MnCO_3$-FPNPs, and $MnCO_3$.

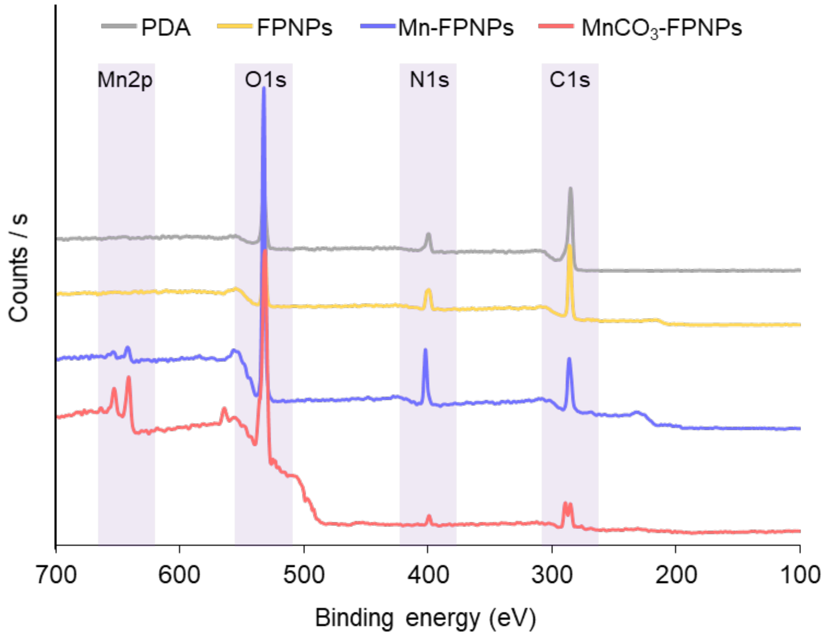

Figure S5. XPS survey scans of control PDA nanoparticles, FPNPs, Mn-FPNPs, and $MnCO_3$-FPNPs.

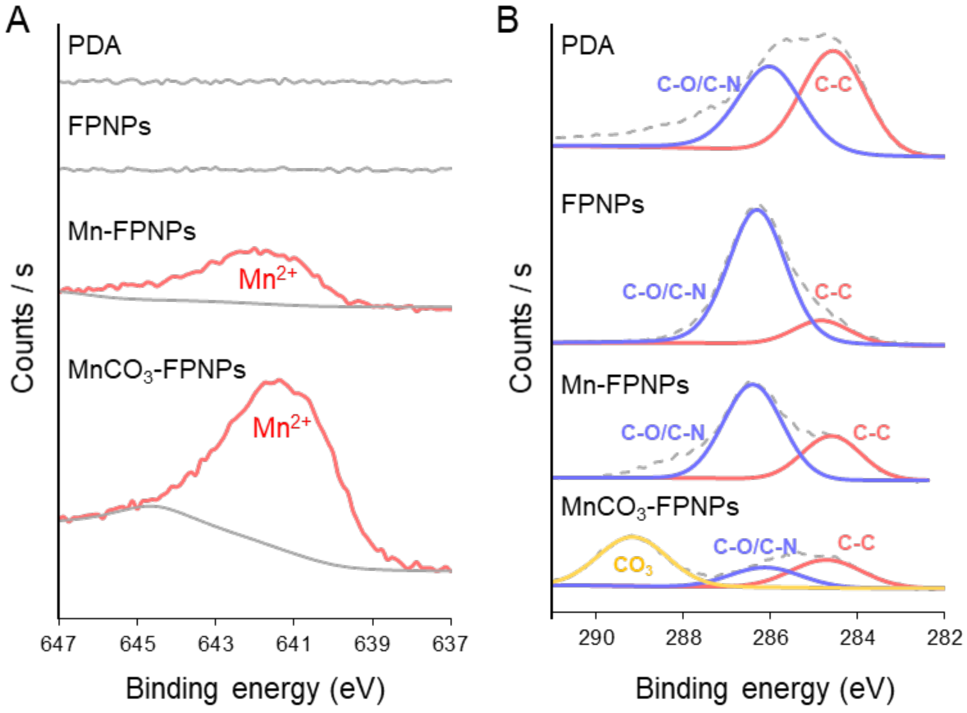

Figure S6. XPS narrow scan spectra of control PDA nanoparticles, FPNPs, Mn-FPNPs, and $MnCO_3$-FPNPs including (A) Mn2p and (B) C1s.

	PDA	FPNPs
Molecular structure	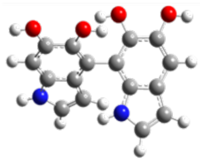	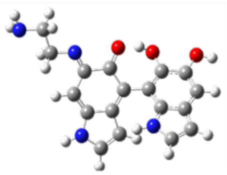
LUMO	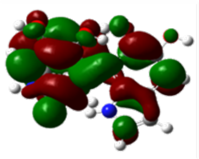 -0.53 eV	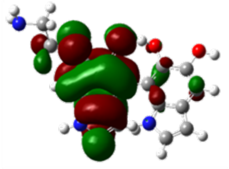 -3.11 eV
HOMO	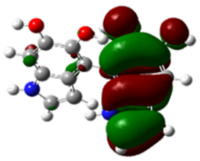 -5.01 eV	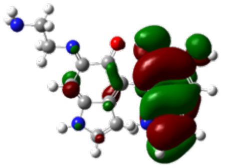 -4.82 eV
Bandgap	4.48 eV	1.71 eV

Figure S7. Theoretical calculated molecular orbitals of PDA and FPNPs using DFT calculations at the B3LYP/3-31G (d,p) level.

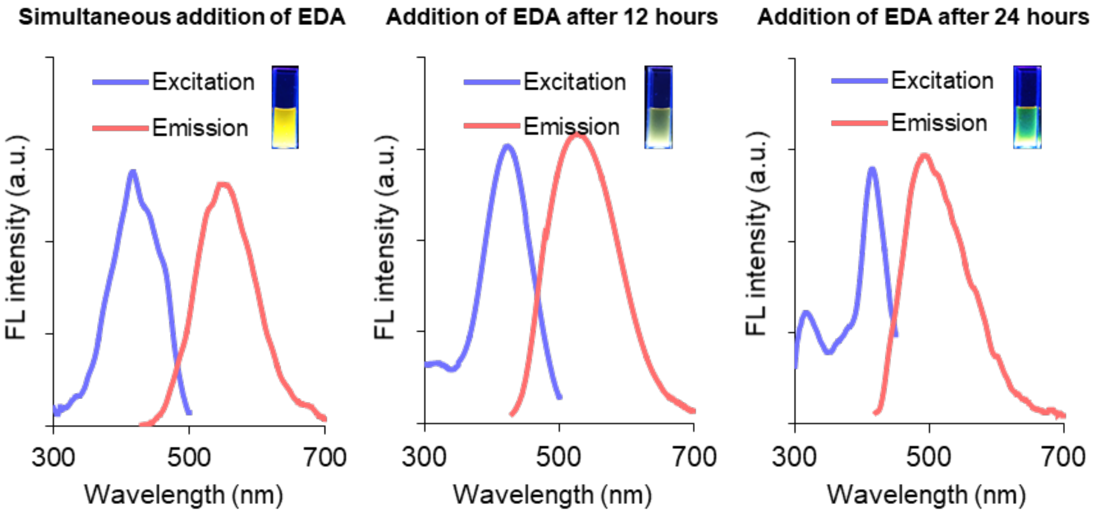

Figure S8. Fluorescence spectra change of FPNPs with EDA addition time.

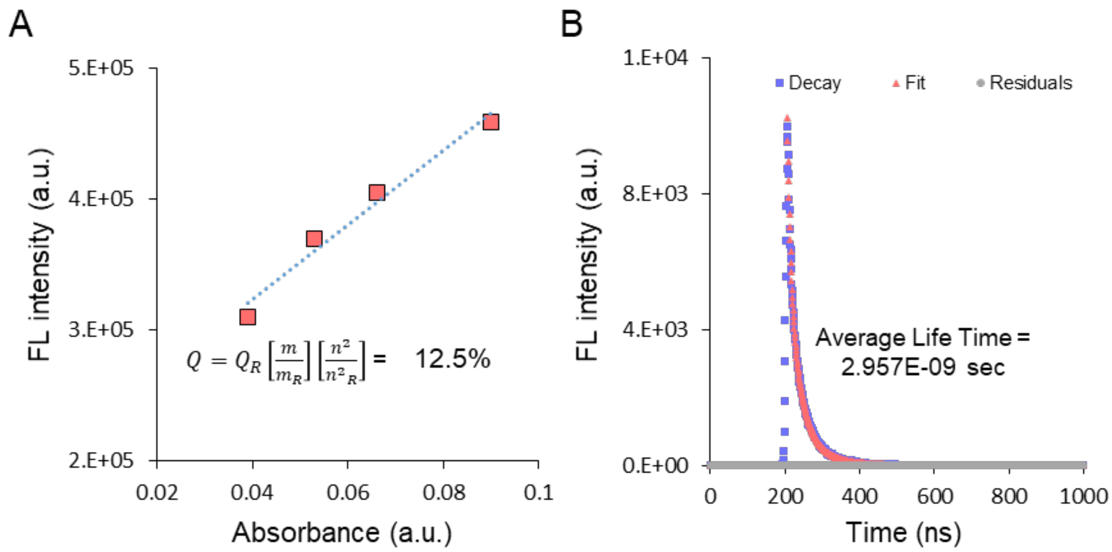

Figure S9. (A) Quantum yield and (B) fluorescence lifetime analysis of FPNPs.

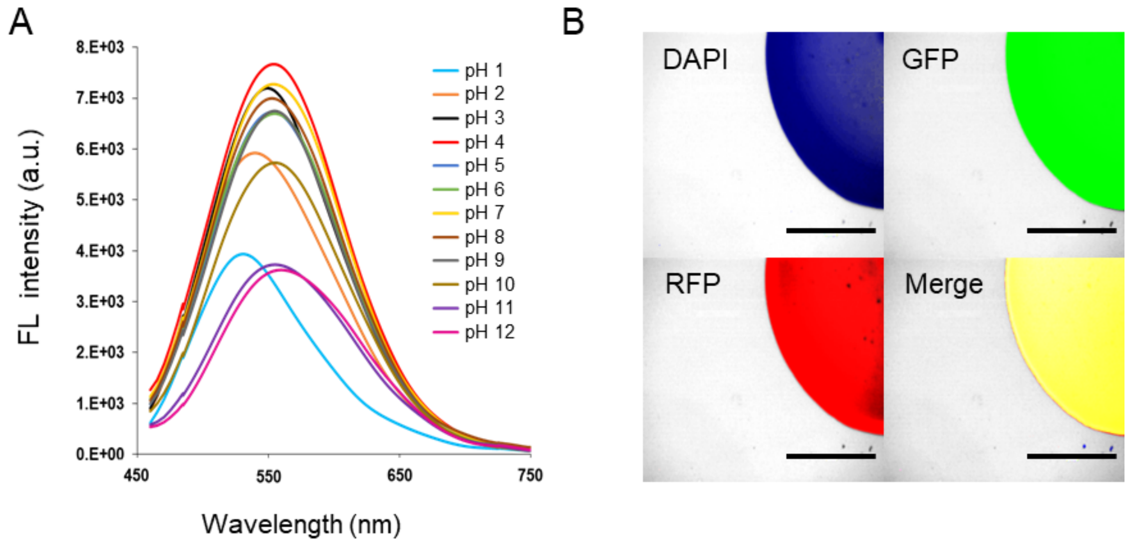

Figure S10. (A) Fluorescence spectra of FPNPs at various pH conditions. (B) Fluorescence images of FPNPs solution using fluorescence microscopy at DAPI, GFP, and RFP field (scale bar = 1000 μm).

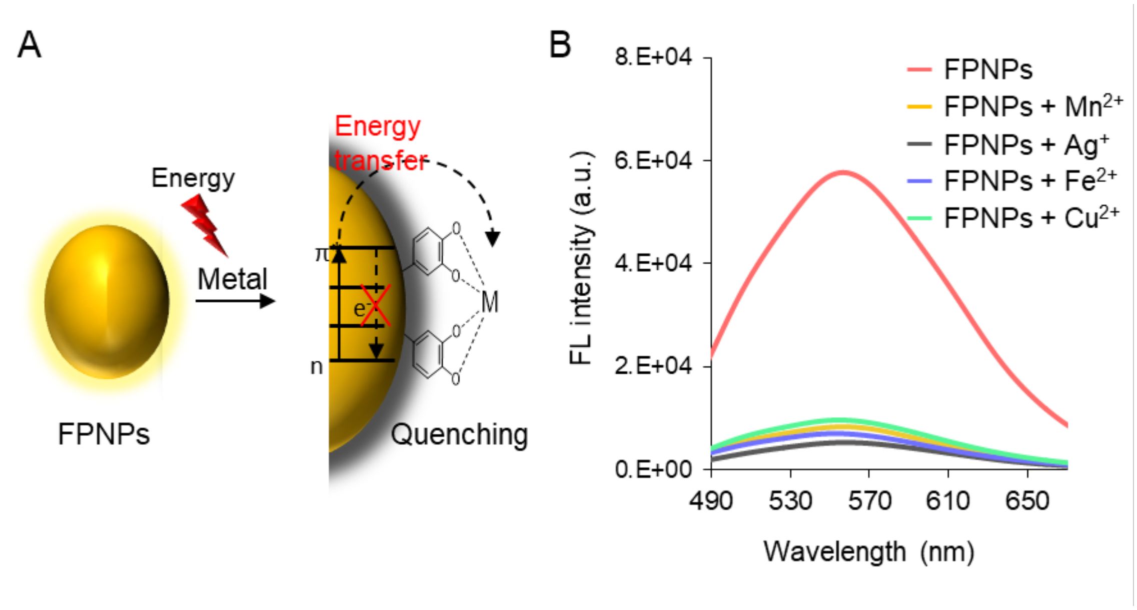

Figure S11. (A) Systematic illustration of the fluorescence quenching mechanism of FPNPs by metal species. (B) Fluorescence quenching spectra of FPNPs in the presence of manganese (Mn^{2+}), silver (Ag^{+}), iron (Fe^{2+}), and copper ions (Cu^{2+}).

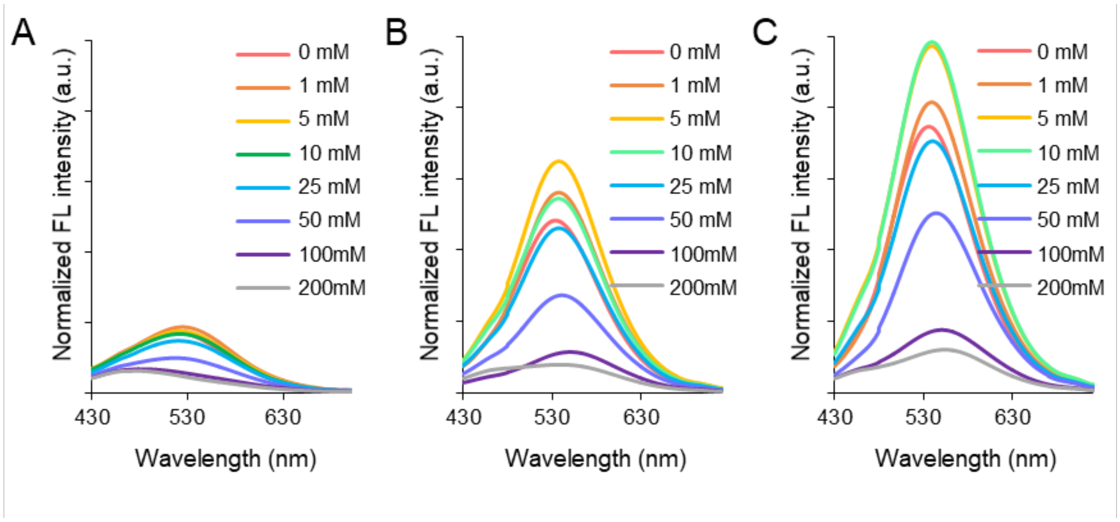

Figure S12. Fluorescence quenching intensities of (A) 0.1, (B) 0.5 and (C) 1 mg/mL of FPNPs solutions with various $MnCl_2$ concentrations.

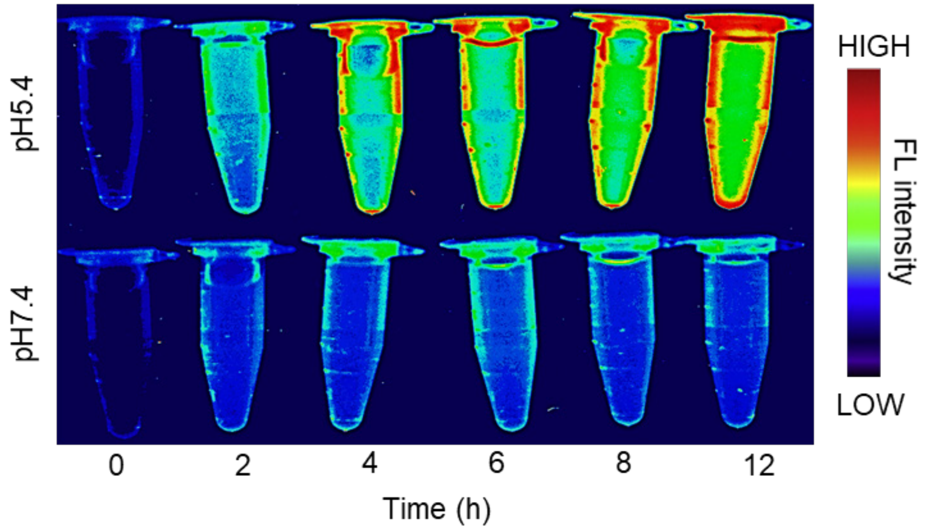

Figure S13. Time-dependent fluorescence images of $MnCO_3$-FPNPs at pH 5.4 and pH 7.4.

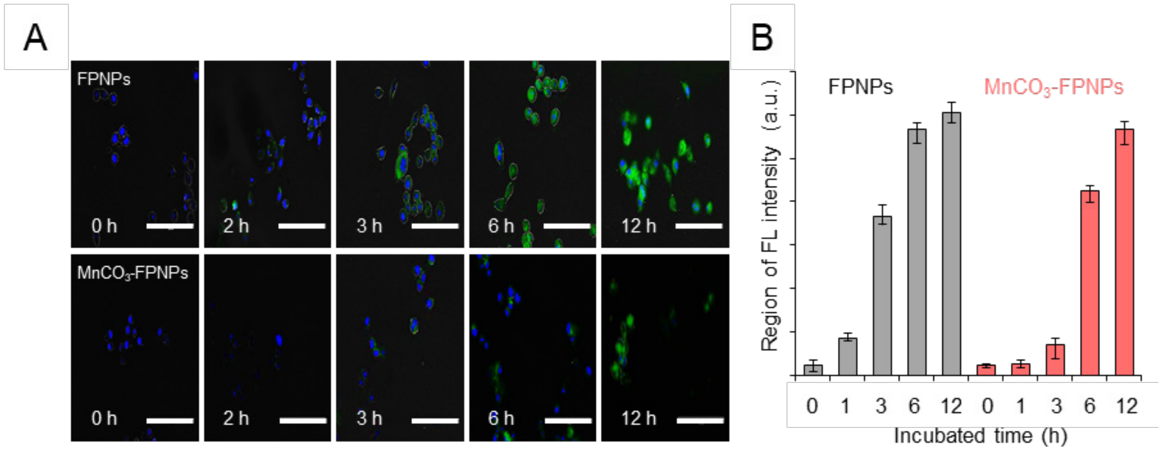

Figure S14. (A) Time-dependent fluorescent images for cellular uptake after treating with FPNPs and $MnCO_3$-FPNPs and (B) their regions of fluorescence intensity (scale bar = 100 μm).

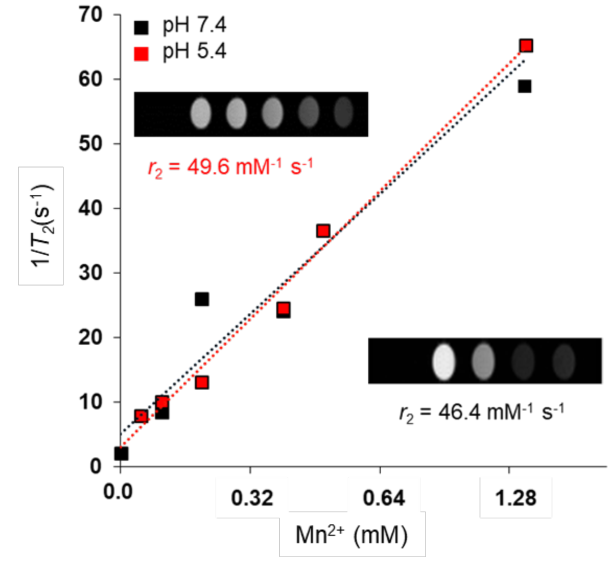

Figure S15. r_2 values of $MnCO_3$-FPNPs at pH 7.4 and pH 5.4.

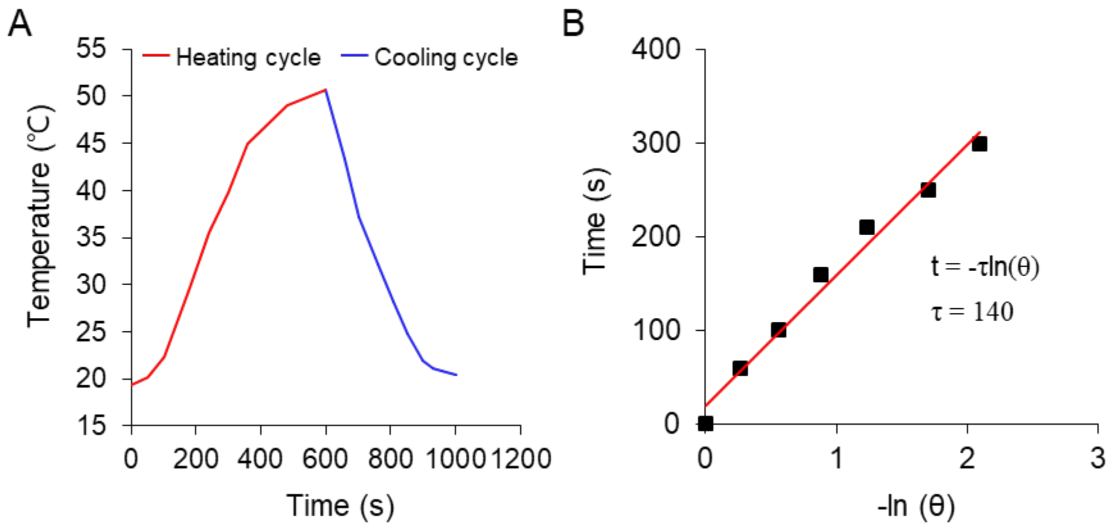

Figure S16. (A) Heating and cooling curves of $MnCO_3$-FPNPs solution with NIR laser irradiation. (B) Linear fit of time/-ln(θ) obtained by the cooling process.

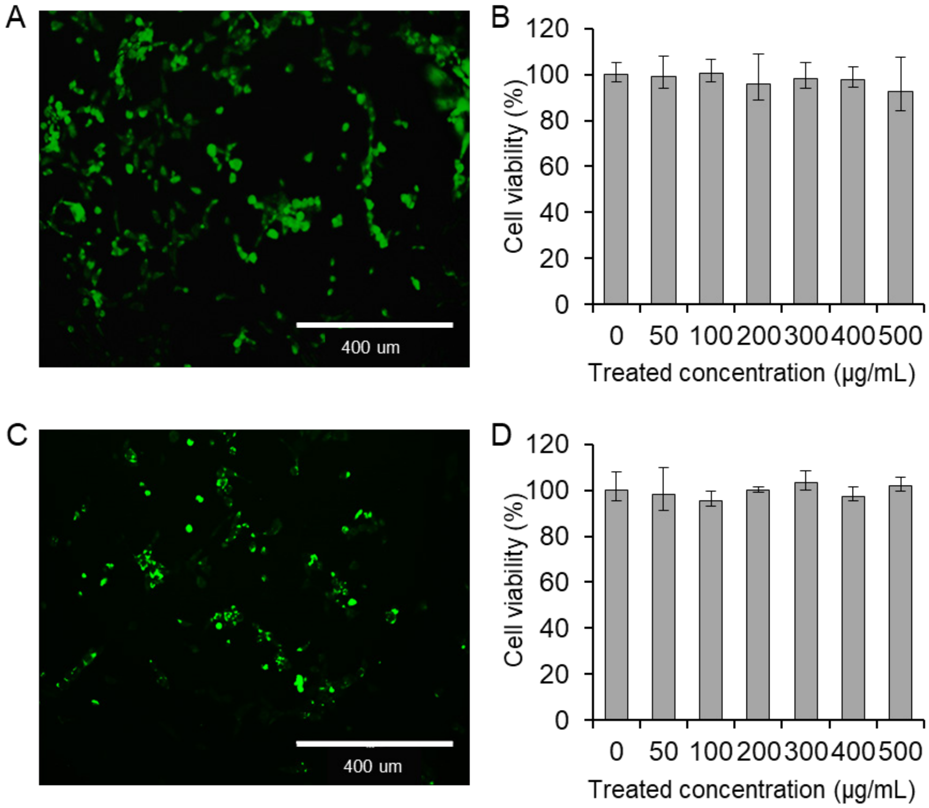

Figure S17. Intracellular fluorescence images of (A) 4T1 cells and (C) MRC-5 cells treated with $MnCO_3$-FPNPs. Cytotoxicity evaluation with various concentrations of $MnCO_3$-FPNPs in (B) 4T1 cells and (D) MRC-5 cells (*n* = 5).

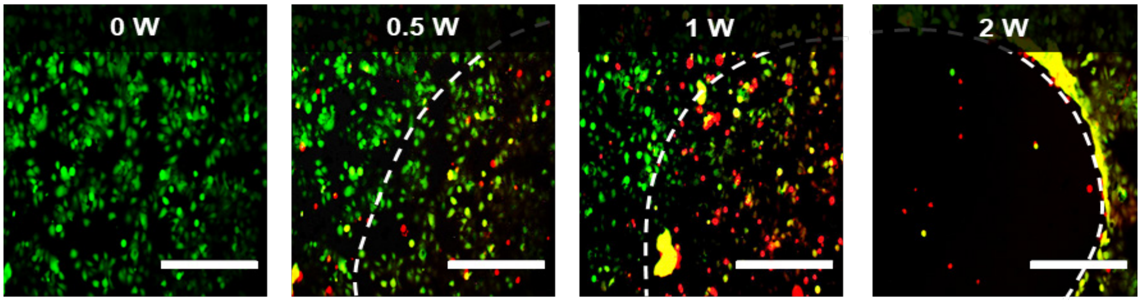

Figure S18. Fluorescence images of 4T1 cells stained with a live-dead kit after treated with $MnCO_3$-FPNPs (0.5 μg/mL) under irradiated by an 808 nm laser at a power density of 0.5, 1, and 2 W cm^{-2} (scale bar = 400 μm).

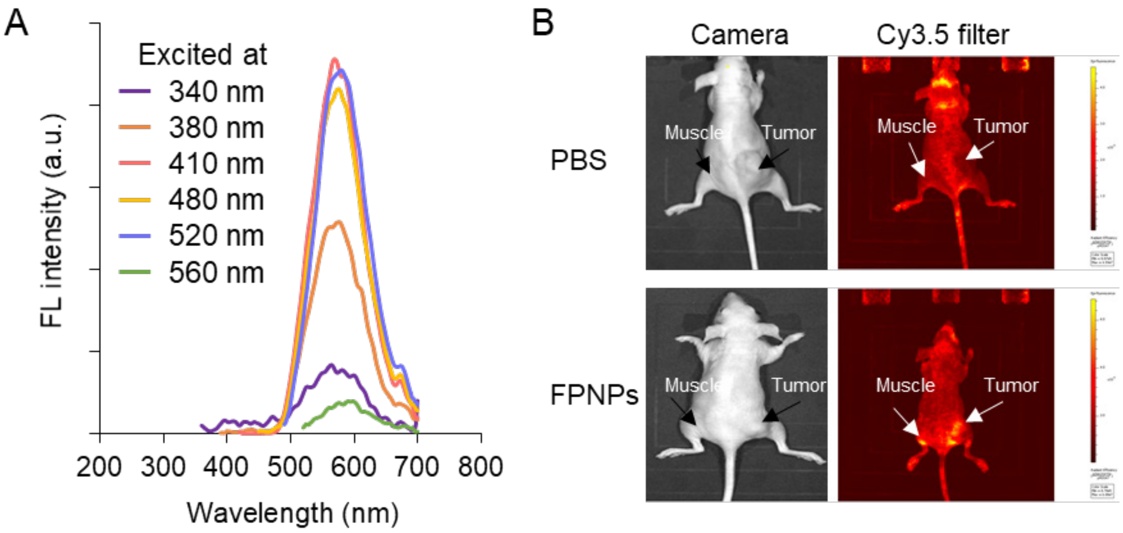

Figure S19. (A) Excitation dependent fluorescence emission spectrum of FPNPs. (B) Fluorescence images of muscles and tumors of balb/c nude mice injected with PBS and FPNPs.

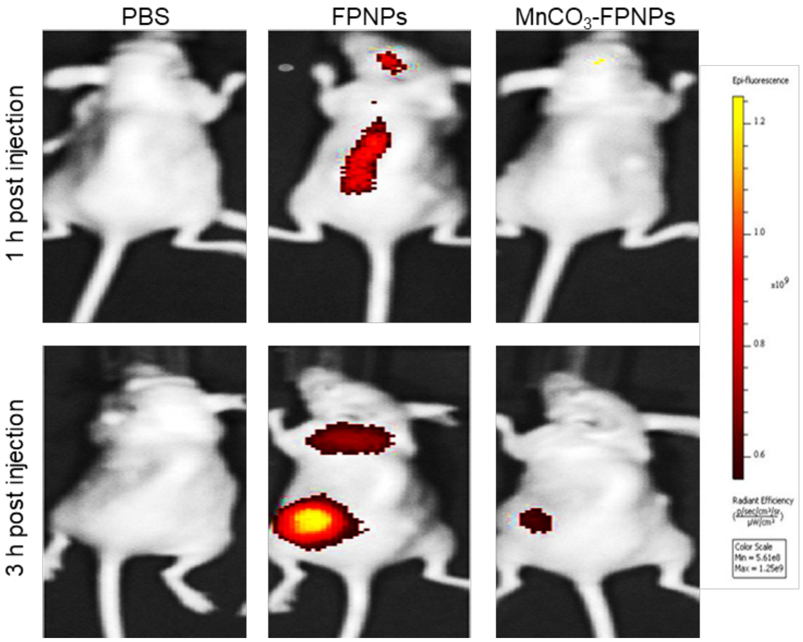

Figure S20. Time-dependent *in vivo* fluorescence imaging of 4T1 tumor-bearing balb/c nude mice after intravenous injection of PBS, FPNPs, and $MnCO_3$-FPNPs.

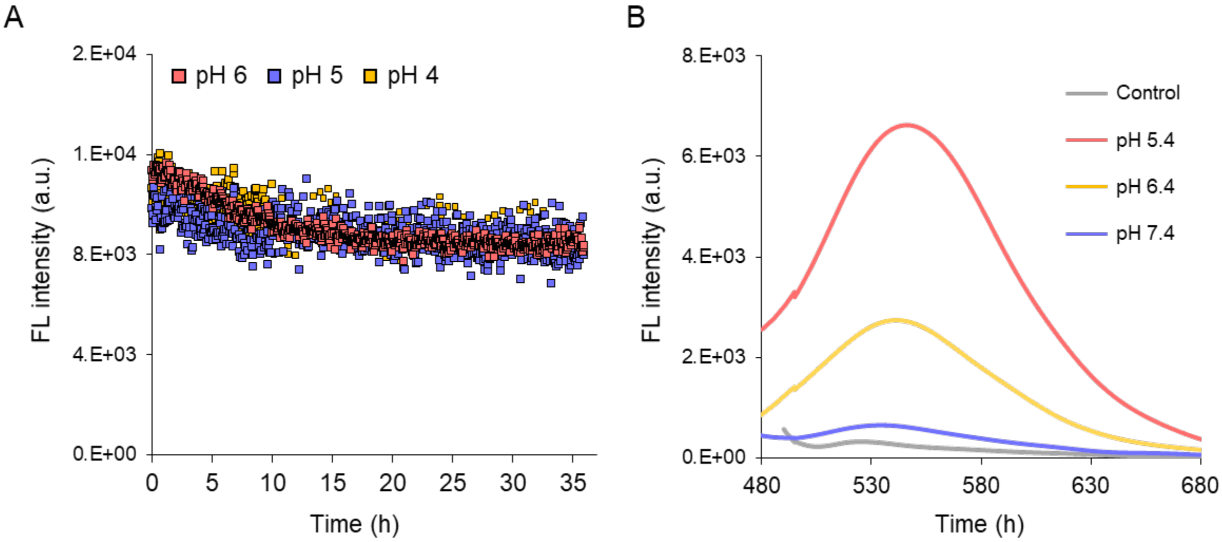

Figure S21. (A) Photostability of FPNPs at acidic conditions for 35 h. (B) Fluorescence change of $MnCO_3$-FPNPs after 3 h in pH 5.4, pH 6.4 and pH 7.4 solutions.

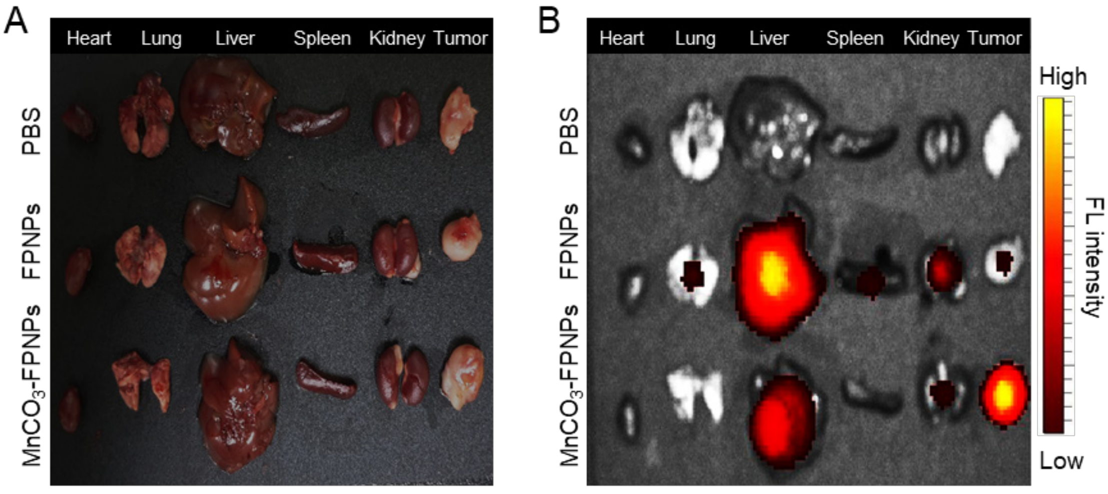

Figure S22. (A) Tissue biodistribution analysis of 4T1 tumor-bearing mice treated with PBS, FPNPs, and $MnCO_3$-FPNPs at 24 h after injection and (B) their representative fluorescence images from IVIS.

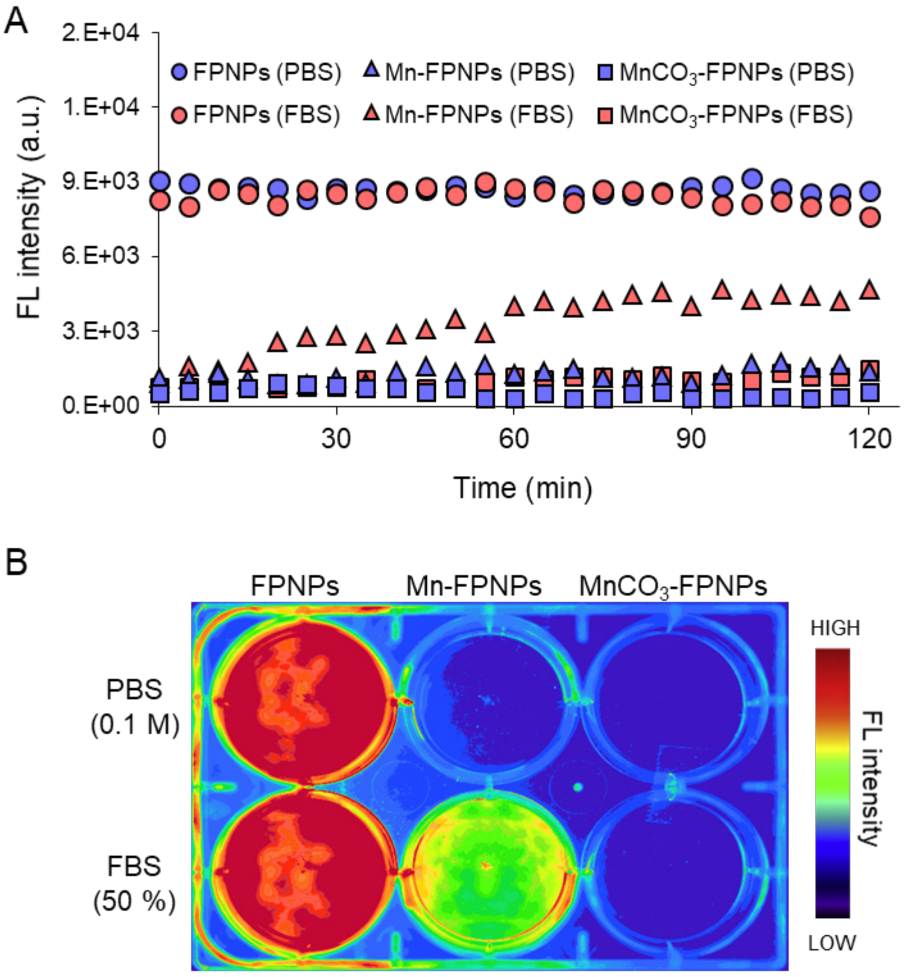

Figure S23. (A) Time-dependent fluorescence intensities of FPNPs, Mn-FPNPs, and $MnCO_3$-FPNPs in PBS (0.1 M) and FBS-containing PBS solution (FBS 50%). (B) Fluorescence images of FPNPs, Mn-FPNPs, and $MnCO_3$-FPNPs immersed in PBS and FBS-containing PBS solution after 2 h.

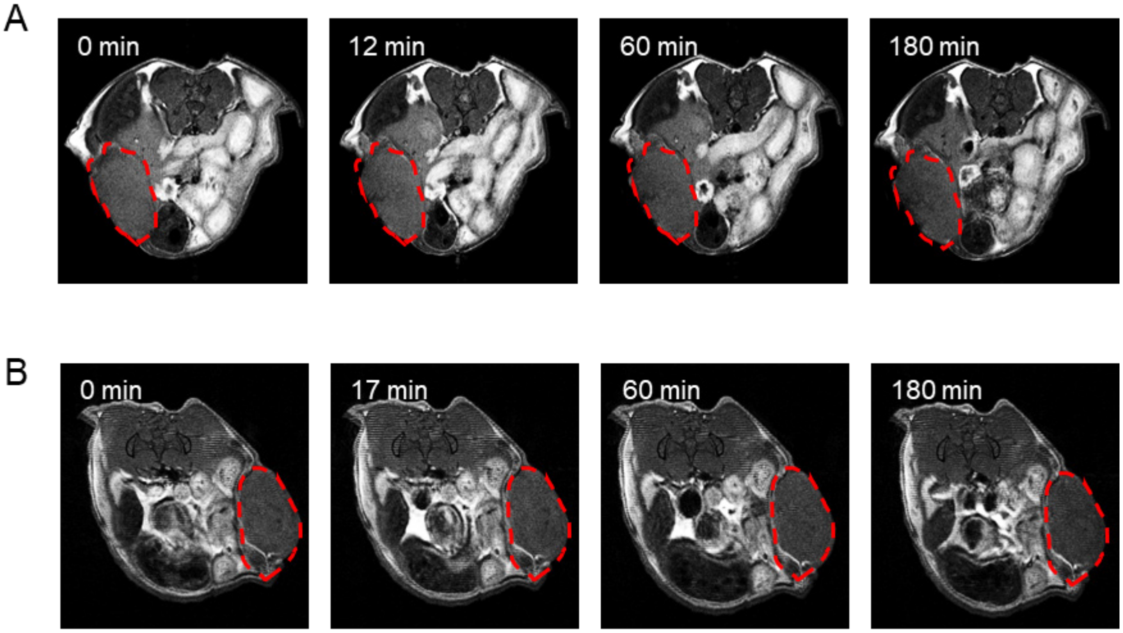

Figure S24. Time-dependent T_1-weighted MR imaging of 4T1 tumor-bearing balb/c nude mice injected with (A) PBS and (B) FPNPs.

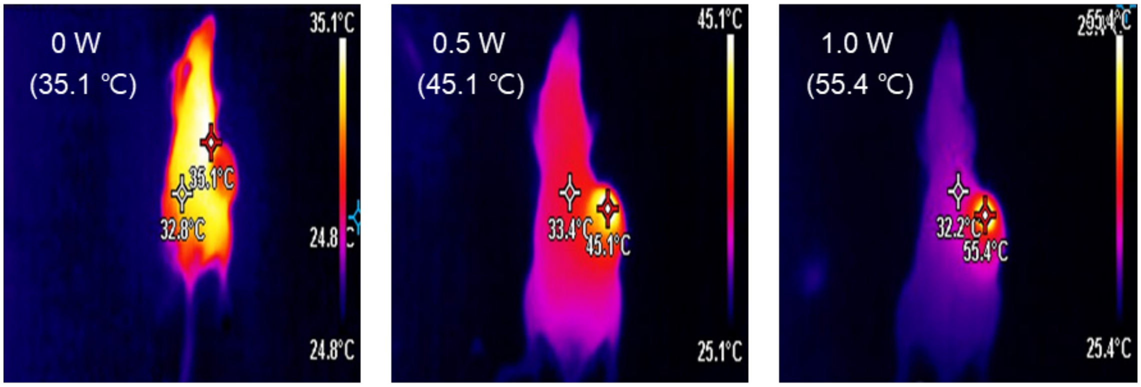

Figure S25. IR thermal images of 4T1 tumor-bearing balb/c nude mice with $MnCO_3$-FPNPs injection according to the power densities of 808 nm laser.

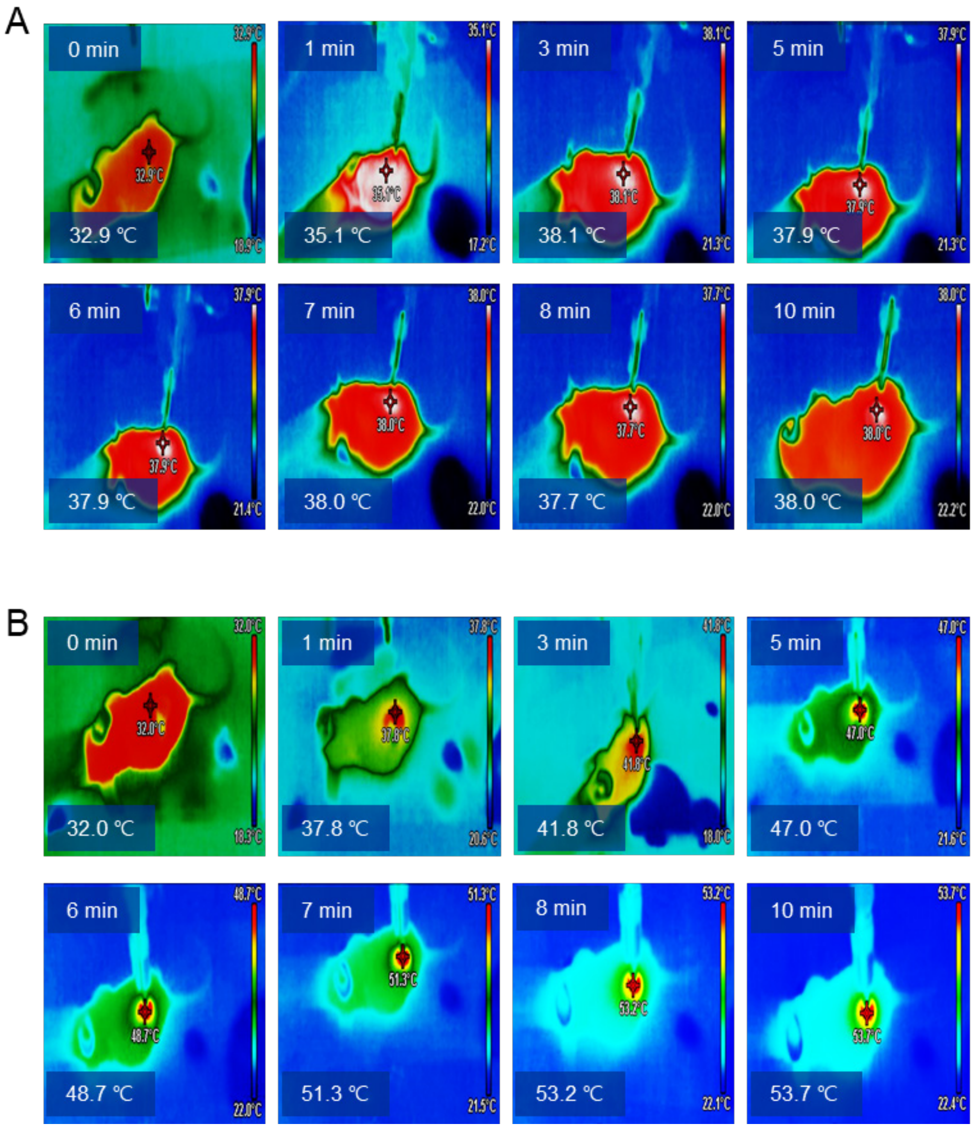

Figure S26. Time-dependent IR thermal images of 4T1 tumor-bearing mice with (A) PBS and (B) $MnCO_3$-FPNPs injection under exposure to the 808 nm laser (1.0 W cm^{-2}) for 10 min.

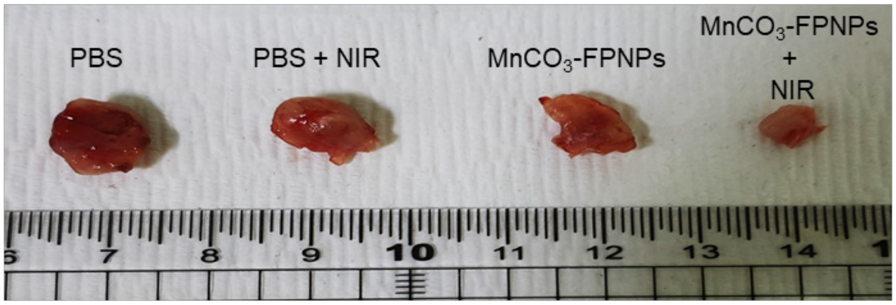

Figure S27. Excised tumors image taken from 4T1 tumor-bearing balb/c nude mice of various groups with different treatments after 14 days.

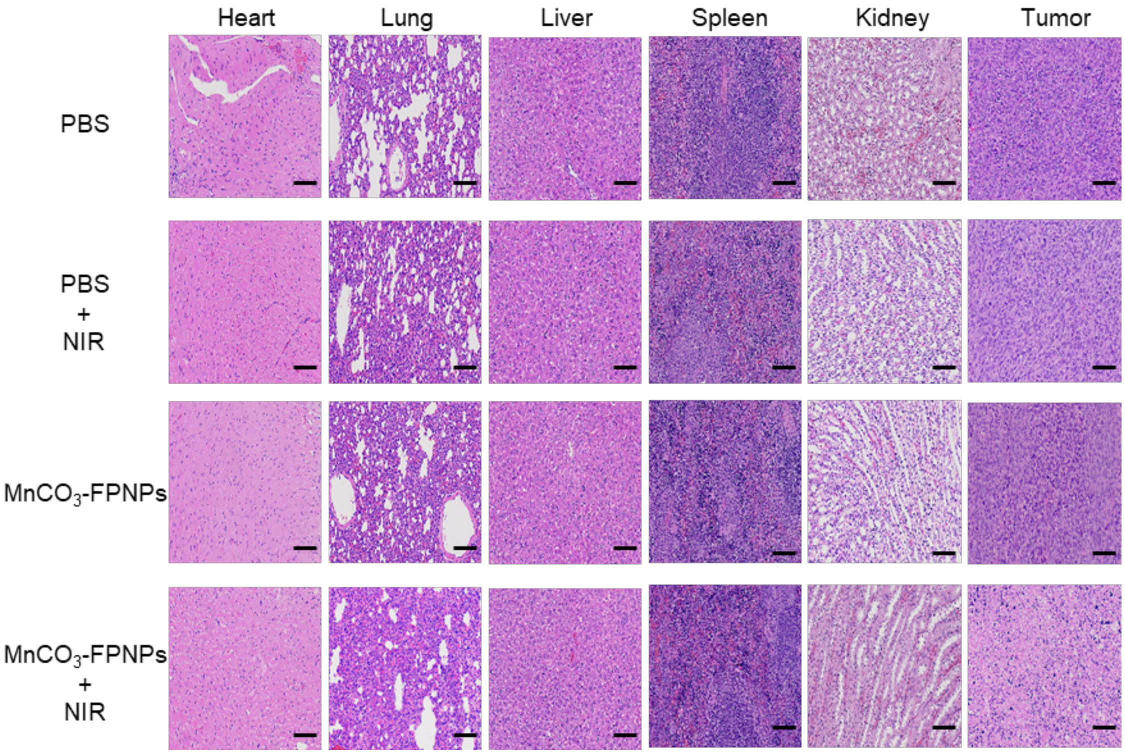

Figure S28. H&E stained images of heart, lung, liver, spleen, kidney, and tumor obtained from 4T1 tumor-bearing balb/c nude mice of PBS, PBS + NIR, $MnCO_3$-FPNPs, and $MnCO_3$-FPNPs + NIR treated groups after 14 days (scale bar = 50 μm).
